# Structure-Forming Potential of Plant Components in the Reformulation of Composite Films Produced from Citrus Pectin and Vegetable Purée

**DOI:** 10.3390/molecules31132318

**Published:** 2026-07-01

**Authors:** Monika Janowicz, Magdalena Karwacka, Agnieszka Ciurzyńska, Karolina Szulc, Sabina Galus

**Affiliations:** Department of Food Engineering, Institute of Food Sciences, Warsaw University of Life Sciences, Nowoursynowska Str., 159c, 02-776 Warsaw, Poland; magdalena_karwacka@sggw.edu.pl (M.K.); agnieszka_ciurzynska@sggw.edu.pl (A.C.); karolina_szulc1@sggw.edu.pl (K.S.)

**Keywords:** edible films, composite, edible packaging, citrus pectin, vegetable purée

## Abstract

This study investigated the rheological, structural, barrier, mechanical, optical, and thermal properties of composite edible films based on citrus pectin and vegetable purées derived from broccoli, cauliflower, pumpkin, carrot, and their blends. Film-forming formulations were characterized in terms of rheological behavior, thickness, microstructure, gas and water vapor permeability, optical and mechanical properties, water contact angle, and thermal stability. The incorporation of vegetable purées significantly modified the properties of the pectin-based matrices. All film-forming solutions exhibited non-Newtonian shear-thinning behavior, with flow behavior index values below unity. The addition of vegetable purées markedly increased viscosity and flow resistance, indicating the formation of more structured systems with stronger intermolecular interactions. Apparent viscosity increased from 0.19 Pa·s in the control sample to 1.41 Pa·s and 1.19 Pa·s in the broccoli (B) and broccoli–cauliflower (B-CF) formulations, respectively, while the consistency coefficient increased from 0.29 to 51.38 Pa·s^*n*^. Composite films exhibited lower water contents (0.090–0.114 gH_2_O·g_d.m._^−1^) than the control film (0.179 gH_2_O·g_d.m._^−1^) and were thicker (170–282 μm) than the pure pectin film (125 μm). Barrier analysis revealed a reduction in water vapor permeability from 18.99·10^−10^ to 10.74–14.69·10^−10^ g·m^−1^·s^−1^·Pa^−1^ and a decrease in carbon dioxide permeability from 21.95 to 10.47–17.91 GRT. The carrot-containing film exhibited the highest tensile strength (62.17 MPa), whereas the pumpkin–carrot film demonstrated the most favorable combination of barrier and mechanical properties, including the lowest oxygen permeability (6.95 GRT), low water vapor permeability (10.74·10^−10^ g·m^−1^·s^−1^·Pa^−1^), and high tensile strength (51.02 MPa). Thermogravimetric analysis revealed similar three-stage degradation profiles for all samples, while vegetable incorporation modified moisture release and increased residual mass. The obtained results confirmed the research hypothesis that vegetable-processing by-products can serve as valuable structure-forming components of pectin-based composite films and that interactions between vegetable-derived biopolymers and citrus pectin improve the mechanical, barrier, and functional properties of the resulting materials. Among the tested formulations, the pumpkin–carrot film demonstrated the greatest potential for further development as a biodegradable packaging material. The utilization of vegetable by-products in pectin-based films represents a sustainable approach supporting circular economy principles and the development of environmentally friendly packaging systems.

## 1. Introduction

Sustainability is currently a priority for scientists, food technologists, entrepreneurs, and ordinary citizens. It is estimated that over 1 billion tons of food worldwide are lost or wasted annually, equivalent to approximately 1 billion meals per day [1]. Global food waste is also estimated to account for 8–10% of total greenhouse gas emissions, with fruits and vegetables having the highest waste rate of all food products—nearly half of all fruits and vegetables are thrown away. Some of the losses occur during the preparation of fruits and vegetables for consumption, and waste is the unfit parts, the percentage of which depends on the type of raw material [2]. In this way, according to the data, up to 5% of the initial mass of green beans, 16% of potatoes, 19% of carrots, and even up to 46% of the mass of pineapple and 66% of green peas is lost [3,4]. However, in the case of fruit and vegetables, losses occur at every stage of the supply chain—from agricultural production, through storage and processing (losses), to distribution and consumption (waste). This amount varies both overall and at individual stages of the supply chain, depending on the region—its level of industrialization, climatic conditions, and social affluence. Industrialized regions such as Europe, North America, Oceania, and the industrialized part of Asia struggle not only with raw material losses at the agricultural production and post-harvest sorting stages, stemming from the high quality standards set by their retail buyers (chain stores). Fruit and vegetable waste at the end of the supply chain is also a significant problem. Approximately 15–30% of consumer purchases are wasted. On the other hand, in developing countries, losses in agricultural production and at the post-harvest and distribution stages prevail, which is associated with financial, managerial and technical constraints in areas such as harvesting, storage in difficult climatic conditions, infrastructure, packaging and marketing systems [5]. The above facts are confirmed by the FAO report from 2021 [6], which was a breakthrough in understanding global food waste in the retail, foodservice, and household sectors. It revealed greater than expected availability of food waste data, particularly at the household level, and showed that food waste generation per person in households was more consistent globally than previously thought [1]. In the context of a circular economy, the management of high-value by-products generated during vegetable processing is particularly important. The production of frozen foods, concentrates, purées, juices, and ready-made meals generates significant amounts of so-called technological waste, which includes vegetables that do not meet commercial requirements in terms of shape, size, or appearance, as well as parts of the raw material removed during peeling, cutting, grinding, and calibration. Although this material often does not reach retail, it retains high nutritional and functional value, making it an attractive raw material for non-food applications and the production of biodegradable packaging materials. Currently, there are six waste management strategies, called the waste hierarchy: prevention, minimization, reuse, recycling, energy recovery, and disposal [7,8]. Among the various ways of managing overproduction, seasonality, and perishability of fruits and vegetables, such as their distribution to those in need [9], extraction of specific compounds [10] or biogas production [11], the use of fruits and vegetables as a matrix-forming ingredient for the production of edible films and coatings for various purposes seems to be a very interesting and promising trend in sustainable development [2,4,12]. Particularly interesting vegetables include pumpkin, broccoli, cauliflower, and carrots, whose production and processing on an industrial scale generate significant amounts of by-products. Frozen food plants reject, among other things, broccoli and cauliflower floret fragments, stems, leaves, vegetables with non-standard dimensions, and mechanically damaged parts of the raw material. In the case of carrots, waste is generated primarily during peeling, calibration, and cutting, while pumpkin processing generates significant amounts of pulp that does not meet commercial requirements, as well as residue from cutting out specific shapes. The choice of these vegetables as packaging components is justified by their chemical composition. Carrots are a valuable source of pectins, cellulose, hemicelluloses, and carotenoids, primarily β-carotene. Pectins and dietary fiber demonstrate the ability to form a continuous polymer matrix, while carotenoids can act as natural antioxidants that enhance the bioactive activity of the film. Pumpkin contains significant amounts of cell wall polysaccharides, pectins, starches, and carotenoids, making it a valuable structural component and a source of functional substances that enhance the protective properties of materials. Broccoli and cauliflower, belonging to the cruciferous family, are rich in dietary fiber, cellulose, hemicelluloses, and pectins, which can participate in the formation of the polymer network of films. Furthermore, they contain phenolic compounds, glucosinolates, and their transformation products with documented antioxidant and antimicrobial properties. The presence of these components allows for the production of active packaging materials capable of limiting oxidation processes and the growth of undesirable microflora on food surfaces. Another significant argument for using carrot, pumpkin, broccoli, and cauliflower varieties is the natural presence of various biopolymers. Pectins, cellulose, hemicelluloses, and small amounts of proteins and starches can interact to create more stable and flexible structures than materials based on single components. This allows us to reduce the amount of additionally added polymers and leverage the natural potential of plant-based raw materials to create biodegradable films and edible coatings. Managing by-products generated during the processing of pumpkins, carrots, broccoli, and cauliflower aligns directly with the principles of sustainable development and the waste management hierarchy. Instead of diverting high-quality waste to animal feed, composting, or energy production, it can be used to produce materials with higher added value. This approach contributes to reducing food losses, increasing the efficiency of plant-based raw materials, and developing innovative, biodegradable alternatives to conventional plastic packaging [3,12,13].

Edible films and coatings, for example, wax coatings on citrus fruits or Yuba—an edible film based on cooked soy milk—have been used for centuries to protect food from water loss, thus extending its shelf life and giving it an attractive shine. These practices occurred many years before the molecular mechanisms of film matrix formation and edible coatings were understood and continue to this day. However, these materials only became the subject of wider scientific interest in the 1950s. The terms film and edible coating are often treated as synonyms. However, there is a significant difference between them. Although they can be obtained from solutions with the same formula, the technique used to form them differs. Edible films are structures with sufficient cohesion and mechanical strength, ensuring their formation outside the food product, for example, in Petri dishes. Edible coatings are formed directly on the surface of the food product, e.g., by immersing it in a film-forming solution [12,13,14]. Due to its potential as a method for extending the shelf life of food products, as an innovative alternative to gluten-containing products such as tortillas, lavash, or pancakes in a gluten-free diet, or as a novel alternative to sandwich wrap, baking bags, or tea bags, and in the future, perhaps even to plastic packaging, the concept of edible films and coatings is currently attracting enormous scientific interest. However, numerous studies indicate the need for further research into the assessment of the mechanical properties (elasticity and tensile strength), thermal properties, optical properties (color and opacity), wettability, and morphology of edible films, as these characteristics significantly impact the functional properties and functionality of the structures studied. The above-mentioned properties depend on several parameters related to the composition of the matrix-forming solution, the conditions of its preparation (solvent, pH, concentration, temperature), and the type of additives used (cross-linking agents, antimicrobial agents, plasticizers, emulsifiers) [15]. The structure-forming capabilities of polysaccharide raw materials, such as starch and its derivatives, alginates, chitosan, pullulan, and pectins, as well as lipid (waxes, acylglycerols, and shellac) and protein (corn zein, gelatin, whey proteins, wheat gluten) as basic components of edible films and coatings have been the subject of numerous scientific reports. The addition of plant and animal ingredients, often with bioactive properties, leads to improved adhesiveness and durability of edible films and coatings during their processing, storage and transport [16]. Therefore, numerous studies are being conducted to improve the formulation of matrix-forming solutions, resulting in laminated or composite materials that combine the advantages of individual components and minimize their disadvantages [17,18,19,20,21], active materials in which the matrix includes antimicrobial components, in particular essential oils [22,23,24], structures subjected to cross-linking [25] and reinforced with nanocomponents to produce bionanocomposites [26,27]. Another promising trend in research on edible films and coatings, which fits perfectly with the idea of sustainable development, is the addition of fruit and vegetable ingredients to matrix-forming solutions. Fruits and vegetables can serve as multifunctional components of edible films and coatings [28,29,30]. The first studies on fruit and vegetable “packaging” materials were published in the 1990s [13]. Over the past three decades, a number of studies have been published on this topic, demonstrating that, thanks to their rich chemical composition, fruits and vegetables can serve as promising sources of multifunctional ingredients in the formulation of matrix-forming solutions [12]. However, not only the presence but also the interactions between individual components play a significant role in the design of the final materials. Ingredients such as pectins, cellulose, starch, and proteins present in fruit or vegetable pulp demonstrate the potential to create a matrix of biodegradable, renewable, inexpensive, and, importantly, in some cases, edible materials. These materials can provide a good oxygen barrier [31]. It has been shown that the natural combination of several matrix-forming components, lipids, and bioactive components found in fruits and vegetables can help create natural composite structures and overcome the problems of phase separation and brittleness of materials obtained from blends of single components [32,33,34]. For example, in a cellulose medium, pectins can form intermolecular bonds between their homogalacturonic regions and cellulose chains. Pectins also counteract the negative effects of plasticizers such as glycerol [35]. Pectins and proteins naturally present in bananas can form bonds between their molecules, improving the mechanical properties of fruit- and vegetable-based materials [36]. Hydrogen bonds between pectin and starch chains can also positively influence the mechanical properties of the resulting structures. The higher the starch content in the matrix-forming solution, the better the mechanical properties of the final material [37]. However, Otoni et al. [12] indicated that materials based solely on fruit and vegetable purées typically lack sufficient barrier properties and, due to their low adhesiveness and mechanical properties, make detachment from the surface on which they were dried difficult. To prevent those issues, combining fruit and vegetable components with pure polymers supporting the formation of integral materials was found beneficial [12,30].

Pectins are hydrocolloids of plant origin. These polysaccharides occur in both the cell walls and the intercellular spaces of land plants, with particularly high levels of pectin found in young fruits and tissues of citrus, bananas, apples, chokeberries, and peaches. Lower amounts are found in carrots, potatoes, tomatoes, and pumpkins [29]. On an industrial scale, pectin is obtained from apple pomace (pectin content of 12–15%) and citrus fruit pomace (pectin content of 20–35%). A characteristic of pectins used in the presented research is their ability to form edible films and coatings [38]. However, pectin films are considered effective barriers to oxygen and lipids. They can also be used in the microencapsulation of flavors, thanks to their barrier against volatile compounds. Although this type of pectin-based structure has a high permeability to water vapor, which is due to the hydrophilic nature of these compounds [30,39].

The scientific goal of this study was to evaluate high-value by-products and by-products generated during vegetable processing, particularly pumpkin, broccoli, cauliflower, and carrots, as components of pectin-based composite packaging films. The research focused on analyzing the potential for utilizing the stabilizing, gelling, thickening, and binding properties of citrus pectin in synergy with naturally occurring biopolymers in these raw materials, such as pectins, cellulose, hemicelluloses, starch, and proteins. These components, present in vegetable by-products, demonstrate structure-forming potential and can serve as a source of bioactive compounds that influence the functional properties of the resulting materials. The scope of the research undertaken in this paper consisted of verifying the hypothesis that by-products and by-products generated during the processing of pumpkin, broccoli, cauliflower, and carrots can constitute a valuable raw material for the production of composite edible films, and that interactions between the biopolymers contained within them and citrus pectin lead to improved mechanical, barrier, and functional properties of the resulting materials. It was also assumed that the use of these raw materials would enable the development of biodegradable packaging materials that align with the concept of sustainable development and a circular economy by utilizing valuable by-products of the vegetable industry.

## 2. Results and Discussion

### 2.1. The Effect of Vegetable Purée on Rheological Properties of Film-Forming Solutions

**Figure 1:**
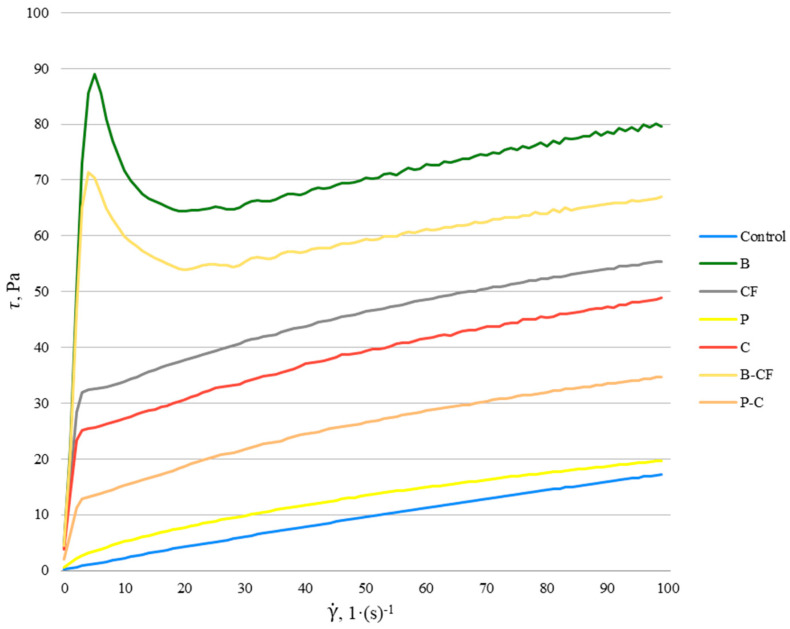
Flow curves of film-forming solutions prepared from vegetable purée and citrus pectin. Variant: Control—corresponds to the control sample containing only citrus pectin. B—sample with added broccoli purée; CF—sample with added cauliflower purée; P—sample with added pumpkin purée; C—sample with added carrot purée; B-CF—sample with added broccoli and cauliflower purée. P-C—sample with added pumpkin and carrot purée.

Rheological techniques are often used as a fundamental tool in process engineering in production facilities and food quality control. They influence the selection of appropriate production parameters and the stability of liquid food products under various storage conditions, making production more efficient [40]. The solution flow curve, i.e., the relationship between shear stress and shear rate, is a key tool for understanding the response of matter to mechanical loading [41]. Based on the conducted experiments, it was found that all tested film-forming solutions with vegetable purées and pectin exhibited increasing shear stress over the course of the study, as shown in Figure 1. Simultaneously, it was observed that solutions with vegetable purées exhibited higher shear stress throughout the entire range of the flow curves than the control solution (Figure 1).

The flow curves of the film-forming solutions are presented in Figure 1, while the rheological parameters obtained from fitting the Ostwald–de Waele model are summarized in Table 1. The incorporation of vegetable purées significantly affected the rheological behavior of the pectin-based film-forming solutions. The broccoli-containing (B) and broccoli–cauliflower (B-CF) formulations exhibited the lowest flow behavior index values (*n* = 0.089 ± 0.008 and 0.092 ± 0.002, respectively), indicating the strongest pseudoplastic behavior among all tested samples [42]. At the same time, these formulations showed the highest consistency coefficients (*k* = 51.38 ± 1.84 and 41.18 ± 3.40 Pa·s^*n*^, respectively), demonstrating the greatest resistance to flow and the most developed internal structure. This observation is consistent with the flow curves shown in Figure 1, where samples B and B-CF exhibited the highest shear stress values throughout the entire shear rate range. The consistency coefficient decreased in the following order: B > B-CF > CF > C > P-C > P > Control. Higher *k* values indicate stronger intermolecular interactions and a more structured network within the film-forming solutions. In contrast, the control sample exhibited the lowest *k* value (0.289 ± 0.012 Pa·s^*n*^), reflecting the lowest resistance to deformation and flow. All tested formulations showed flow behavior index values below unity (*n* < 1), confirming their non-Newtonian shear-thinning character. The highest *n* values were observed for the control sample (*n* = 0.889 ± 0.002) and the pumpkin formulation (*n* = 0.595 ± 0.006), indicating behavior closer to that of Newtonian fluids. The apparent viscosity measured at a shear rate of 50 s^−1^ was highest for samples B (1.408 ± 0.036 Pa·s) and B-CF (1.191 ± 0.069 Pa·s), whereas the lowest value was recorded for the control sample (0.190 ± 0.009 Pa·s). These results indicate that the addition of vegetable purées increased the viscosity and flow resistance of the film-forming solutions, with the most pronounced effect observed for broccoli- and broccoli–cauliflower-based formulations. A sharp increase in shear stress at low shear rates was observed for samples B and B-CF (Figure 1). This behavior is not fully described by the classical Ostwald–de Waele model and is reflected in the lower coefficients of determination obtained for these samples (*r*^2^ = 0.648 and 0.667, respectively) compared with the remaining formulations (*r*^2^ = 0.968–0.999). This may indicate the presence of additional structural phenomena or experimental effects that are not accounted for by the power-law model [43]. To facilitate the interpretation of the rheological parameters presented in Table 1, it should be emphasized that the consistency coefficient (*k*) represents the resistance of a fluid to flow and reflects the strength of intermolecular interactions within the system [44]. Therefore, the high *k* values observed for samples B and B-CF indicate a more structured network and greater resistance to deformation during shearing. Conversely, the low *k* value of the control sample suggests a less structured system with lower flow resistance [45]. The flow behavior index (*n*) describes the deviation of a fluid from Newtonian behavior. A value of *n* = 1 corresponds to a Newtonian fluid, whereas values below unity indicate shear-thinning (pseudoplastic) behavior. The very low *n* values obtained for B and B-CF demonstrate the strongest shear-thinning character, meaning that their viscosity decreases markedly with increasing shear rate. In contrast, the control and pumpkin-containing samples exhibited higher *n* values and therefore behaved more similarly to Newtonian fluids [46].

The apparent viscosity (*η*) measured at 50 s^−1^ provides a practical measure of flow resistance under processing conditions. The highest apparent viscosity values obtained for B and B-CF confirm their highly structured nature and greater resistance to flow, while the control sample exhibited the lowest viscosity. Collectively, the results indicate that the incorporation of vegetable purées, particularly broccoli and broccoli–cauliflower blends, strengthened the internal structure of the film-forming solutions, increased their viscosity, and enhanced their resistance to deformation under shear [46,47].

In summary, the incorporation of vegetable purées into pectin-based film-forming solutions increased shear stress, apparent viscosity, and consistency coefficient values compared with the control formulation. The strongest effect was observed for broccoli (B) and broccoli–cauliflower (B-CF) systems, which exhibited the highest flow resistance and the most pronounced pseudoplastic behavior. All formulations behaved as non-Newtonian shear-thinning fluids, although the degree of shear-thinning varied depending on the type of vegetable purée incorporated into the system.

### 2.2. The Effect of Vegetable Purée on the Chemical Composition of Films

Water content in food is one of the main criteria determining its nutritional value, storage suitability, quality and product’s microbiological stability. Determining water content in a material is primarily used during the food design stage to assess its impact on the quality of the resulting product. The water content in the tested films ranged from 0.090 to 0.180 gH_2_O·g_d.m._^−1^ (Table 2). Studies have shown that adding vegetable purées to the films’ matrix significantly reduces their water content. The film obtained with broccoli purée had the lowest water content compared to the control sample (Table 2). At the same time, literature data indicate that, of the vegetables selected for the purées used in the coatings, pumpkin and carrots, after heat treatment, contain an average of 7.75 g of total carbohydrates per 100 g, including 4.9 and 4.6 g of digestible carbohydrates, respectively [48]. This may also result in higher water content in the prepared and tested films compared to the composite structures obtained from broccoli and cauliflower purées or their mixtures. The presence of water in the composition of edible coatings at a properly designed level ensures appropriate plasticity, which also shapes their functional properties.

One approach to determining the proper storage conditions for food products is the glass transition temperature, which states that determining the humidity and water activity at which a product is physically stable guarantees its storage properties. Both water and simple and complex carbohydrates have been shown to exert a plasticizing effect in edible film matrices through interactions. The glass transition significantly influences the molecular movements of polymers, thus defining the barrier and mechanical properties of edible films. These properties are determined by chemical bonds, such as hydrogen bonds between polymer chains in the edible film matrix [49]. A difference of several percent in water content between samples is crucial in developing the appropriate composition of the finished product. It affects the structure of the film, including its mechanical and packaging properties [41]. The content of basic nutrients (carbohydrates, protein, fat) in the tested vegetable–pectin films was calculated based on the dry matter content and information obtained from food composition and nutritional value tables [48]. The estimated values are given in Table 2. The calculations also took into account that all vegetable films contained, on average, the same amount of carbohydrates from the addition of citrus pectin to support the matrix structure of the resulting composite film, at approximately 5%. Statistical analysis identified three homogeneous groups based on carbohydrate and protein content (Table 2). At the same time, in the case of carbohydrate content, a significant effect of the type of purée introduced into the matrix was demonstrated on the dry matter content of the resulting biopolymers.

The highest carbohydrate content in dry matter was found in the matrices structured with pumpkin purée, carrot purée, and their mixtures. Statistical analysis confirmed the influence of the purée composition on the formulation of the tested films and the potential influence of carbohydrates on the properties of the resulting polymer structures (Table 2), which will help verify the research hypothesis in the performance studies. Statistical analysis also identified three homogeneous groups based on protein content (Table 2). However, due to the absence of protein in the control sample, statistical analysis was also performed excluding this film. In this case, no significant differences were observed between the effect of the vegetable component on the dry matter content of the structured matrix of pumpkin purée with a medium content of the building block component and the carrot, pumpkin-carrot, and cauliflower purées, which, based on the estimated protein contents, were defined as low-protein structure-forming materials. As part of the analysis of the results obtained for estimating the fat content in the tested composite film matrices structured through interactions of vegetable purée components supported by citrus pectin, two homogeneous groups were determined as part of the statistical analysis (Table 2), of which the control sample constituted one independent and significantly different group. Therefore, a film obtained solely from citrus pectin was excluded from detailed analysis, which allowed us to conclude that, despite the trace amounts of fat present in the vegetable purée, all vegetable components and their mixtures affect the dry matter content of the resulting biopolymer matrix (Table 2).

It has been shown that a natural combination of several matrix-forming components, lipids, and bioactive components found in fruits and vegetables can help create natural composite structures and overcome the problem of phase separation and brittleness in materials obtained from blends of single components [33,34]. For example, in a cellulose medium, pectins can form intermolecular bonds between their homogalacturonic regions and cellulose chains. Pectins also counteract the negative impact of plasticizers such as glycerol or water [50]. This article has already mentioned that pectins and proteins naturally occurring in plant tissues can form intermolecular bonds, improving the mechanical properties of fruit- and vegetable-based materials. Hydrogen bonds between pectin and starch chains can also positively influence the mechanical properties of the resulting structures, and the higher the starch content in the matrix-forming solution, the better the mechanical properties of the final material. It has also been shown that biopolymer materials based solely on fruit and vegetable purées are usually characterized by insufficient barrier properties due to too low adhesiveness and mechanical properties that make it difficult to separate them from the substrate on which they were dried [12,28,30].

### 2.3. The Effect of Vegetable Purée on Thickness of Films

The thickness of edible films is a parameter that influences the biological properties and shelf life of coated food. It depends on the properties of the film-forming solution, the production method, and, above all, the components that make up the matrix of the resulting coating. Thickness is a physical quantity that influences many important characteristics of edible films, such as strength, permeability, and appearance. This is crucial for the packaging industry, as even small changes in film thickness can affect its final physical and protective properties [29]. Thickness analysis of the tested composite polymers obtained from citrus pectin and vegetable purée ranged from approximately 125 µm to over 282 µm, depending on the composition (Table 2). This confirmed the findings of Wang et al. [51], who developed edible films based on carrot purée, carboxymethylcellulose, corn starch, and gelatin, with glycerol as a plasticizer. The thickness of the carrot films determined by the authors ranged from approximately 110 to 240 µm and depended on the type and amount of ingredients used. McHugh et al. [13], who were among the first to determine the thickness of films with the addition of various types of fruit purées, determined their values at the following levels for films from peach purée: 186 µm, apricot—192 µm, apple—215 µm, and pear—258 µm. The film thickness results obtained in the study were comparable, falling within the range reported by the authors, and the differences were due to the use of a different type of substance to produce the film-forming solution (low-methoxyl citrus pectin).

### 2.4. The Effect of Vegetable Purée on the Microstructure of Films

Visual assessment of food microstructure provides information about its components, physical properties, and product quality. The photographic documentation presented in Table 3 includes surface images and cross-sections of the tested composite edible films. The attached images show differences between the matrix produced for the composite films, particularly in the control sample, and the other structures obtained from the interaction of vegetable components with pectin. The control film is characterized by the smoothest surface structure and a denser matrix in cross-section compared to the other films. The presence of voids in the structure of the vegetable films is observed, not only in the central portion of the composite matrices but also in the form of surface irregularities (as indicated in the film images by red arrows), which may be the result of insufficient deaeration of the film-forming solutions. Numerous studies are being conducted to improve the formulation of matrix-forming solutions, which results in this research, but also in laminated or composite materials used in practice, combining the advantages of individual components and minimizing their disadvantages [17,21], active materials in which antimicrobial ingredients were included in the matrix [24], cross-linked structures [25], and reinforced with nanocomponents to produce bionanocomposites [27].

In summary, the observed differences in the appearance and thickness of the dry films were directly related to the formulation composition. The addition of various vegetable purées influenced the content of water, carbohydrates, proteins, and lipids in the pectin matrix, which altered its structural and rheological properties. Formulations containing higher amounts of solids, particularly carbohydrates derived from pumpkin and carrots, exhibited higher viscosity and formed thicker films after drying. Conversely, films with broccoli and cauliflower were characterized by lower water content and a more compact structure. The presence of natural plant ingredients also promoted the formation of a stable composite matrix, reducing brittleness and improving the uniformity of the films. Consequently, differences in dry film thickness and visual characteristics are a natural consequence of the differences in solids content and rheological behavior of the tested formulations.

The study described by Peng et al. [52] on the analysis of the structure of pectin films, as well as the studies and examples described, demonstrate that the presence of additives such as vegetable purée, which create composite structures, influences changes in the structure of the matrices, introducing significant irregularities into the structure of the films, which can alter their properties and affect the functional characteristics of the potentially produced packaging materials. It can also be assumed that a natural combination of several matrix-forming components of natural origin, such as polysaccharides, lipids, or bioactive components found in fruits and vegetables, can co-create the structures of composite polymeric materials and overcome the functional problems of these film matrices resulting from their delamination at the interface and their brittleness compared to materials in the form of films or coatings obtained based on a mixture of single components [33,34]. For example, in a cellulose medium, pectins can form intermolecular bonds between their homogalacturonic regions and cellulose chains. At the same time, pectins also counteract the negative impact of the presence of plasticizers, such as glycerol [50].

### 2.5. The Effect of Vegetable Purée on the Water Vapor, Oxygen and Carbon Dioxide Permeability

The main purpose of food packaging is to protect the product from external factors and maintain its consistent physicochemical properties over time. The most common qualitative changes occur in the proportions of its chemical components, such as water vapor and gases like oxygen and carbon dioxide, which migrate relatively easily to or from the surrounding environment. The barrier properties of packaging are influenced not only by the material from which it is made but also by the product stored in it [53]. Water vapor permeability in the food packaging industry is a crucial indicator, as it determines the ability of edible films to maintain the freshness of stored goods. Reduced water vapor permeability through the film can extend the shelf life of food products stored in edible films. The environment directly influences the water vapor permeability, which is related to the number of hydroxyl groups (-OH) on the molecule. Higher relative humidity and low temperature increase the index, while the opposite parameters decrease it [54,55].

**Figure 2:**
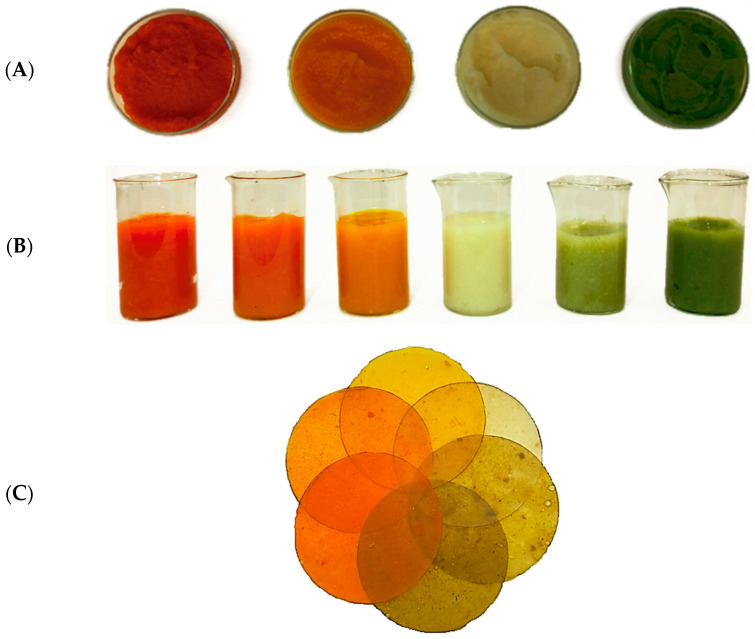
Selected vegetable purées (**A**) and film-forming solutions (**B**) obtained from vegetable purée and citrus pectin; from left: broccoli (**A**,**B**), broccoli-cauliflower (**B**), cauliflower (**A**,**B**), pumpkin (**A**,**B**), pumpkin-carrot (**B**), carrot (**A**,**B**); (**C**)—Composite polymer structures in the form of edible films obtained based on vegetable purée and citrus pectin.

Analysis of the gas and water vapor permeability results showed that four homogeneous groups can be distinguished in terms of oxygen permeability, clearly indicating that the coating made from broccoli purée structured with citrus pectin has the highest permeability at approximately 39 GRT, while the film made from pumpkin-carrot purée has the lowest, approximately 7 GRT (Table 4). At the same time, considering the estimated carbohydrate content of the vegetable purée used as a component supporting the formation of the biopolymer matrix (Table 2), it was found that the content of these compounds did not significantly affect oxygen permeability. However, in the case of carbon dioxide, the polymer matrix made solely from citrus pectin exhibited significantly higher permeability than the other films (∼21.95 GRT) (Table 4). Furthermore, the introduction of vegetable purée into the polymer matrix, regardless of the estimated carbohydrate content, significantly reduced the permeability of the tested films, a desirable feature for packaging materials with potential barrier properties. Additionally, statistical analysis revealed no significant effect of carbohydrate component content on CO_2_ permeability for the vegetable purées and their mixtures (Table 4). The barrier properties of the tested composite materials to water vapor ranged from approximately 10.7 to approximately 19·10^−10^ g·(m·s·Pa)^−1^. Statistical analysis confirmed a significant effect of carbohydrate content on this feature of the tested films. The control film matrix, prepared solely from citrus pectin, exhibited the highest water vapor permeability among the structured film matrices based on vegetable purées and pectin. This also confirms the trend observed for CO_2_, that the barrier properties of the tested composite materials to water vapor significantly increase with the addition of vegetable components, and the carbohydrate content may determine this feature with the increasing amount of compounds in the purée incorporated into the polymer matrix (Table 4, Figure 2A,B).

The permeability of a polymer matrix is a result not only of the interactions of the constituent components of the film-forming solutions but also of the size of the gas molecules. A carbon dioxide (CO_2_) molecule is comparable to an oxygen (O_2_) molecule in size, but is slightly larger and heavier. The effective diameter of a CO_2_ molecule is approximately 3.3 Å, while for O_2_ it is approximately 2.9 Å. Furthermore, a CO_2_ molecule is heavier than an O_2_ molecule; the molar mass of CO_2_ is 44.01 g·mol^−1^, while that of O_2_ is 32.00 g·mol^−1^. Compared to larger molecules, such as those in carbohydrates, proteins, or nucleic acids, it is significantly smaller. A carbon dioxide (CO_2_) molecule is also larger than a water vapor molecule (H_2_O—18.015 g·mol^−1^). The difference in mass is due to the molecular structure: CO_2_ consists of one carbon atom and two oxygen atoms, while H_2_O consists of one oxygen atom and two hydrogen atoms. Therefore, a CO_2_ molecule is larger and heavier than an H_2_O molecule. A water vapor molecule is only one ten-millionth of a millimeter (0.0000001 mm), which also explains why substances completely impermeable to water are relatively easily permeated by water vapor. Pectin films are not as strong as films obtained from other hydrocolloids. However, pectin films are considered an effective barrier against oxygen and lipids. Thus, it should be remembered that such pectin-based structures are highly permeable to water vapor, which results from the hydrophilic nature of these compounds [39]. The control film was characterized by the highest water vapor permeability, and the highest water content was also determined in its composition (Table 2), which, like glycerol, also acts as a plasticizer, determining the hydrophilic/hydrophobic nature of the tested structure, which can modify the mechanism of adsorption–desorption processes of water molecules during storage of products protected with biopolymers obtained as a result of reformulation of pectin films using vegetable and/or fruit purées [56]. As previously mentioned, water vapor permeability depends on the internal structure of each biopolymer matrix, as confirmed by the compact microstructure observed in the control film (Table 3).

Introducing components in the form of vegetable purées and their blends into the matrix of the tested films led to the formation of more porous, less compact, and more uniform microstructures in the composite films (Table 3). The differences in the composition of the tested films may alter the mechanisms by which individual components interact with each other and with the water present in the structure or with water vapor in the environment. Water reacts with pectin through hydrogen bonds [57]; as a result, water cations (H_2_) interact with the overall negative charge of pectin, hydroxyl groups of glycerol and negative charges of compounds present in vegetable purées (carboxyl groups), while anions (O^+2^) connected by positive charges remain available to interact with the hydrogen of the water molecule. The presence of hydrogen bonds hinders water migration, which, in turn, is retained within biopolymer structures, thereby modifying the effect of hydration. However, water permeability cannot be influenced by water retention. It is likely that when the sorption sites in the films are occupied, other water molecules that move under the influence of the pressure difference can be easily transported because there are no adsorption sites where water could be retained. In the case of films containing vegetable purée, the differences in permeability were not statistically significant (Table 4) and ranged from about 10.7 to about 14.7·10^−10^ g·(m·s·Pa)^−1^. The effect differentiating the significance of the effect of the type of vegetable purée used was the carbohydrate content in its composition, as shown by the results of the statistical analysis presented in Table 4 (the effect of carbohydrate content in the composition of the vegetable–pectin composite films on permeability). This effect was attributed to the increased water adsorption capacity of the more porous biopolymer matrices obtained from vegetable purées with the addition of citrus pectin, resulting from the interaction of pectin with water and monosaccharides, which act as water binders. Nevertheless, more flexible and plastic structures were obtained, as demonstrated below by the description of mechanical properties [58].

### 2.6. The Effect of Vegetable Purée on the Optical Properties of Films

The sense of sight plays a key role in assessing product attractiveness, so testing is essential to verify optical properties such as color, its intensity, and transparency. Color, as a subjective experience, most profoundly influences consumer impressions and decision-making when purchasing [59]. Color assessment and perception (Figure 2A–C) of the tested composite edible films were determined by the type of purée used to create the biopolymer matrix. The CIE L*a*b* parameter results may be useful during the design phase of edible films, which may potentially constitute an integral protective coating for ready-to-eat food products. Furthermore, as evidenced by the properties of the structural components used, the composite polymer matrices produced were characterized by chromatic coordinates described in Table 5. The pectin-based film had the highest brightness. At the same time, the biopolymer matrix produced from cauliflower purée had the closest brightness to the control sample. Nogueira et al. [60] examined the effect of blackberry pulp on the visual characteristics of pectin films and demonstrated a significant dependence of the fruit additive on changes in color and characteristics of the resulting film matrix. Compared to the control sample without fruit addition, a decrease in brightness was observed, and depending on the percentage of additional fruit, an increase in the absolute color difference was observed. Results supporting the described trends in changes in optical properties, based on changes in chromatic coordinates, were obtained by Otálora González et al. [61], who studied the characteristics of pectin films with the addition of red beetroot and cabbage. The effect of the vegetable powder was significant due to the properties of the plants in terms of their color, which also determined the obtained color of the vegetable–pectin matrix, similar to the studies performed in this work (Figure 1, Table 5). The fillers used by Otálora González et al. [61] contained betalains or anthocyanins and were rich in non-cellulosic carbohydrates. Based on the results, they were effectively incorporated into the pectin matrix, resulting in a red color change. Therefore, these films would be promising for the development of packaging with active color.

The practical application of packaging produced from colored films is shown in Figure 2 for freeze-dried mini kiwi fruit and beetroot slices. It seems that the possibilities of using this type of packaging are promising in terms of other than just visual values of their functional features, which is confirmed by the results of research by other scientists [62,63,64]. Brito et al. [65] obtained edible films from solid residues generated during the preparation of an isotonic drink, including both fruits (sweet orange, passion fruit, watermelon) and vegetables (zucchini, lettuce, carrots, spinach, mint, sweet potatoes, cucumber, arugula). The films were homogeneous, yellowish, and plastic, highly water-soluble, and, when enriched with pectin, demonstrated very good functional properties. These films, with a small addition of pectin, showed better color, mechanical, and barrier properties, and significantly lower hygroscopicity. Bhargava et al. [66] reported research on the use of dyes derived from food waste, such as blueberry pomace, black rice bran, grape skin, and turmeric residue, as alternatives to synthetic dyes used in colored edible films. Combining the functions of biopolymers with those of bioactive dyes enables the production of biodegradable, intelligent packaging. Kurek et al. [67] used anthocyanin dyes derived from blueberry and red grape pomace in the production of edible packaging. They showed high antioxidant activity and sensitivity to pH changes, which are desirable for colorimetric indicators of food quality. In another study reported by Nisar et al. [68], pectin films enriched with thinned young apple polyphenols were produced, and the authors also reported a decrease in L* values and an increase in a* and b* values. In another study by Sganzerla et al. [69] in the case of films with pine seed starch and pectin with the addition of *Acca sellowiana* extracts, lower L* values and higher a* and b* parameters were also found.

Optical property analysis revealed that the appearance of the tested composite films was closely related to the composition of the formulations used. CIE *Lab* color parameters confirmed the influence of the type of vegetable purée on the brightness and color characteristics of the resulting coatings. The pectin-based control film exhibited the highest brightness, while the addition of purées resulted in a decrease, with the cauliflower matrix exhibiting values most similar to the control sample. The observed color changes can be attributed to the presence of natural pigments found in plant materials, which were effectively incorporated into the biopolymer matrix and imparted characteristic visual characteristics to the coatings.

These results are consistent with previous observations regarding the effect of formulation composition on the structure and properties of the films. In addition to natural pigments, the content of carbohydrates, proteins, lipids, and water from the vegetable purées played a significant role, influencing the organization of the pectin matrix and the final appearance of the coatings. Differences in uniformity, transparency, and color intensity therefore reflected both the chemical composition and the interactions of the formulation components.

It should also be emphasized that the observed differences in dry film thickness are a natural consequence of the varying solids content and distinct rheological properties of the individual formulations. Systems richer in dry components exhibited greater viscosity during application and left a thicker film after drying, which could further affect color perception, opacity, and the overall visual assessment of the resulting coatings.

### 2.7. The Effect of Vegetable Purée on the Mechanical Properties of Films

The primary function of packaging is to protect the product from external factors. A film may be suitable as packaging after passing strength tests to verify its mechanical properties, such as resistance to deformation or external loads [70]. The strength test results for the films ranged from approximately 27 MPa to over 62 MPa, depending on the composition (Table 6).

The lowest strength and highest elongation were observed in the control film. The matrix structure of the control sample was characterized by a tensile strength of approximately 27.4 MPa and an average elongation of over 20% (Table 6). This sample also had the highest water content (Table 2). This is due to the absence of a vegetable feedstock, which acts as a binder and strengthens the film structure. This relationship was confirmed by Yuan et al. [71] who investigated the properties of pectin- and nut-based composite polymers. The structure of the composite film based on carrot purée (C) withstood the highest overload of approximately 62 MPa. The biopolymer obtained from cauliflower purée was the least susceptible to elongation, achieving an elongation of only about 10% of its length. Pereira et al. [72] obtained identical results for pectin films. The reduced stretching capacity can be attributed to the increased number of intermolecular cross-links between pectin molecules and vegetable purée in the composite matrix. Statistical division of the analyzed films into homogeneous groups revealed three groups with respect to strength and four with respect to elongation (Table 6). At the same time, it is interesting to confirm the possibility of practical use of the obtained biopolymers by preparing heat-sealable packaging for finished freeze-dried products, as shown in Figure 2. Therefore, it can be concluded that the tested vegetable–pectin films were characterized by confirmed thermoplasticity and good mechanical properties, despite significant variation in the values of strength and elongation coefficients determined by the types of vegetable purée used. Compared with the composite films described in this study, those described in the literature were characterized by higher tensile strength, which may better meet the requirements for food packaging [30,55,73]. The increase in tensile strength was attributed to the cohesion of polymer chains [56]. Hydrogen bonds formed as a result of the interaction of components [45], in this case all polysaccharides present in the matrix created from a combination of vegetable purée and citrus pectin [36], facilitated the regular arrangement of the matrix chains of the films, creating a sufficiently compact structure despite the presence of irregularities and pores, as in the control sample (Table 3), which influenced and improved its mechanical strength and allowed for maintaining appropriate extensibility and enabled weldability [29].

In summary, the observed differences in appearance, color, thickness, and mechanical properties of the tested films were a natural consequence of the varying formulation compositions. The presence of natural pigments was responsible for the changes in color parameters, while differences in solids content and rheological behavior influenced the film thickness. Simultaneously, interactions between the plant components and pectin led to the formation of stable composite structures with good mechanical properties, confirming their potential for use as biodegradable packaging materials.

### 2.8. The Effect of Vegetable Purée on the Water Contact Angle

Terms like hydrophilicity, hydrophobicity, and wettability are widely used in the literature on all types of films and membranes. They describe the behavior of fluids as they spread on a solid film surface. While wettability is a general term used to describe the spreading behavior of any liquid on a surface, the terms hydrophilicity and hydrophobicity refer to the attraction and repulsion of a surface toward water, respectively [74]. The contact angle is the angle at the interface between water, air, and a solid, and its value is a measure of the probability of water wetting the surface. A biopolymer is considered hydrophilic when its contact angle is less than 90°, whereas values above 90° indicate hydrophobicity [75]. The study conducted is of great importance in the field of packaging. The data obtained allows us to determine whether edible film will function as good packaging [76]. Fogging is a common phenomenon during the storage of packaged food. This is the process of water condensation during refrigerated storage. This, combined with edible film lacking appropriate hydrophobic properties, can completely lose its protective properties [62]. At the same time, it is important to remember that a water droplet wets the surface matrix much more quickly, resulting in a lower contact angle on the smoother side of the resulting vegetable–pectin films. The lack of an uneven surface with varying particle sizes of the vegetable feedstock increases the hydrophilic properties of the films through greater water-to-surface contact [75]. Therefore, due to the absence of the vegetable feedstock, which influences the surface sorption phenomenon [77], the control film exhibited the highest, identical wetting angle on both sides of the obtained structure (Table 7), which is related to the similar surface documented by SEM (Table 2). Research by Çavdaroğlu et al. [78] confirms this phenomenon. The pectin film exhibits the lowest viscosity (Table 1); therefore, water molecules, despite extensive contact with the surface, did not wet the film as thoroughly as in the other cases. The films containing pumpkin and broccoli had the lowest wall angle (Table 7). The combination of the two vegetables negatively affected the film, reducing its hydrophobicity. The same effect occurred for the pumpkin and carrot samples, although on a smaller scale. The flattest droplet appeared on the film with cauliflower. At the same time, the introduction of vegetable purée into the matrix structure can be viewed as an expansion of the interfacial contact surface formed between the tested films and water molecules. In this case, the introduction of purée can be treated as the introduction of a surfactant, which, depending on its amount in the matrix, can determine the hydrolyte-hydrophobic properties of the resulting biopolymer matrix [14,57].

At appropriately selected concentrations, surfactants adsorb at one or more interphase boundaries in the system and significantly alter the work required to widen them. Surfactants typically act by reducing interfacial free energy, although they are sometimes used to increase it. The structure of vegetable purée incorporated into the matrix, consisting of both phyophobic compounds with negligible affinity for the solvent and lyophilic compounds with opposite properties, allows the resulting biopolymer matrices to be characterized as amphiphilic structures, characterized by simultaneous hydrophilic (lyophilic) and hydrophobic (lyophobic) properties. Amphiphilic structures are typically rich in compounds with long-chain molecules that possess groups soluble in one solvent at one end and groups soluble in a second solvent at the other. This causes the molecules to spontaneously form monolayers at the interface (at the point of contact between the two solvents or on the surface of one of them), and within the volume of one of the solvents, they form spherical aggregates (micelles), in which one end of the molecules (the soluble one) points outward and the other toward the center of the sphere. Most amphiphilic compounds used in practice have hydrophobic (“water-hating”) and hydrophilic (“water-loving”) ends, but this phenomenon is not limited to systems containing water [57,79]. Amphiphilicity is a biologically and technologically important phenomenon, as it enables lipids to form cell membranes and to enable certain enzymatic transformations. This technology enables the creation of stable colloidal compounds, such as emulsions, which are later used as dyes, cosmetics, and natural biopolymer packaging materials, among other things. The phenomenon of amphiphilicity is also responsible for the formation of lyotropic (solvent) liquid crystal phases (mesophase) [80], which are characterized by the ability to flow, typical of a liquid, and at the same time by long-range ordering of the molecules constituting it, similarly to the more ordered structures as in crystals, which gives the possibility of research into more complex connections between the matrix components of biopolymer phases and their solvents and plasticizers [81].

Overall, the results demonstrated that the type of vegetable purée significantly influenced the wettability of the pectin-based films. The observed differences in contact angle values indicate that each vegetable purée modified the surface characteristics of the films in a distinct manner. These variations can be attributed to differences in the chemical composition of the vegetables, including the proportions of hydrophilic and hydrophobic constituents, as well as their effect on the microstructure and roughness of the film surface. Consequently, the incorporation of vegetable purées altered the balance between hydrophilic and hydrophobic interactions at the film–water interface, resulting in different wetting behaviors among the tested formulations.

### 2.9. The Effect of Vegetable Purée on the Sorption Properties of Edible Films

**Figure 3:**
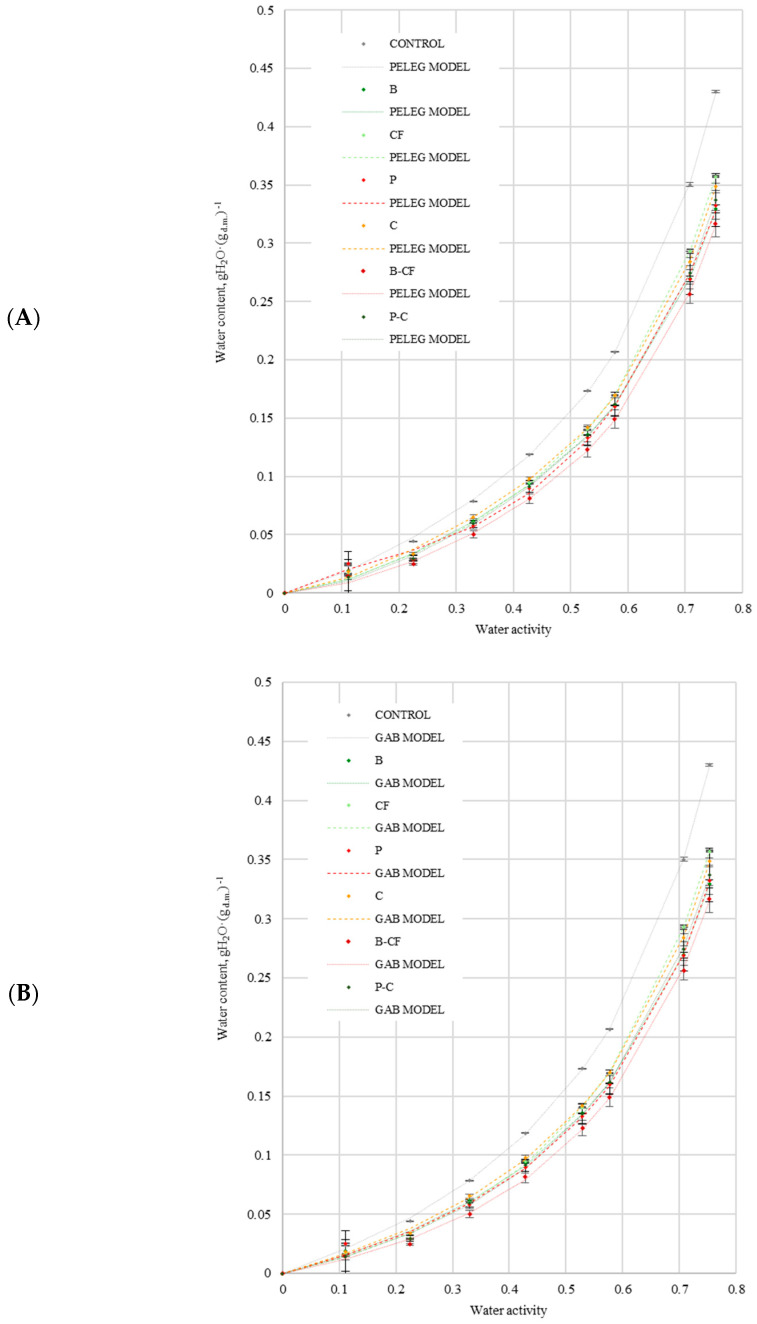
Sorption properties of composite films obtained from vegetable purée with citrus pectin described by the models: (**A**)—PELEG; (**B**)—GAB.

Diffusivity resulting from moisture movement in the environment of natural biopolymers, including packaging materials, is related to water content and activity, as well as to the porosity of materials whose structures are not perfectly smooth and uniform. The mechanism of change is also determined by the type of interactions between the components of the biopolymer matrix. These properties are of great importance during the storage of food products protected by these materials, as the barrier properties of packaging are an inherent functional feature, and the resulting increase in material moisture content can degrade quality characteristics and even compromise the safety of the protected food [82]. Biopolymer composites produced from plant-derived components are diverse in terms of chemical composition (Table 2) and matrix structure, which are visible both on the surface and in the cross-section of the obtained composite films (Table 3). Therefore, their water absorption capacity under constant-temperature conditions was determined experimentally and described using the GAB (Figure 3A) and PELEG (Figure 3B, Table 8). Three criteria were used to evaluate the fit of each isothermal model: mean relative error (MRE), standard error of estimate (SEE), and residual sum of squares (RSS). An MRE value of less than 10% indicates a correlation that provides a good representation of the data. The SEE value accounts for the number of constants in the model and provides a measure of the model’s reliability in describing the experimental data, regardless of the number of parameters. The lower the calculated MRE and SEE values, the better the models’ ability to represent the experimental data. The residual sum of squares (RSS) is important in nonlinear regression, and the fitting procedure is designed to minimize it. These statistical parameters are widely used as a basic criterion for selecting the best equation representing the sorption behavior of materials. It was found that regardless of the type of vegetable purée introduced into the pectin matrix, the presence of the component reduced the water content adsorbed by the tested films from the surrounding environment of a specific water activity (Figure 3). At the same time, statistical analysis showed that a significantly different adsorption mechanism occurs in films formed by interactions between the components structuring the matrices of combined broccoli-cauliflower and pumpkin-carrot purées.

### 2.10. The Effect of Vegetable Purée on the Thermal Properties of Edible Films

**Figure 4:**
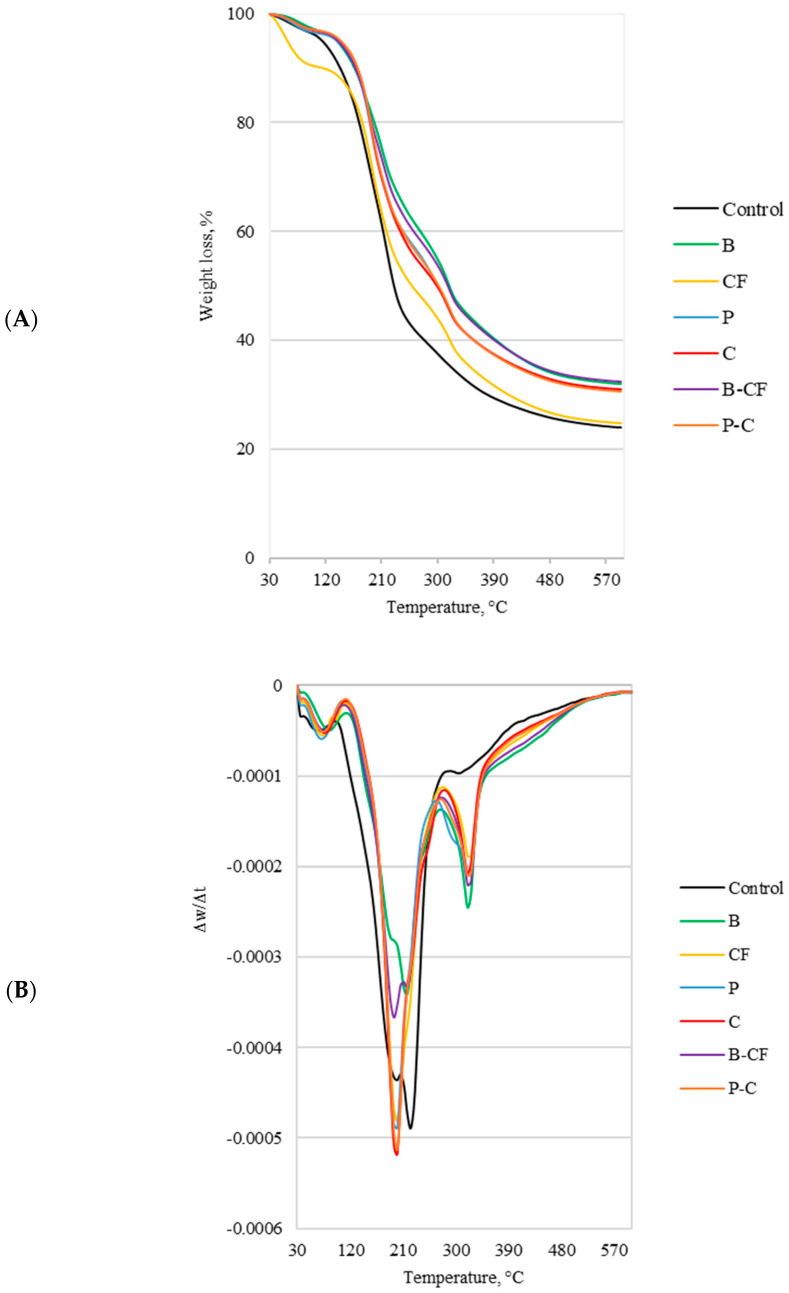
Thermogravimetric analysis of composite films with vegetable purée and citrus pectin results presented as (**A**)—weight loss and (**B**)—dTG curves.

The pectin films containing vegetable purées were tested for their behavior during thermal decomposition. Results of the thermogravimetric analysis are shown in Figure 4, which shows the weight change and weight loss rate over time during heating from 30 to 600 °C, and in Table 8, the peak decomposition temperatures and percentage of weight loss at specific decomposition stages are presented. It should be noted that the temperatures discussed in this section correspond to the maxima of the DTGA curves (T_max_), representing the temperatures of the highest mass-loss rate during thermal degradation, and should not be interpreted as glass transition temperatures (T_g_). Based on the decomposition rate curve, three main mass-loss stages were defined as follows. The first was from 30 to 100 °C, the second from 100 to 270 °C, and the third from 270 to 600 °C, with a peak around 319 °C. Additionally, all curves exhibited a relatively similar pattern, suggesting comparable behavior under heating.

The first stage represents a release of moisture and volatile compounds. The temperature corresponding to the maximum rate of mass loss (T_max_) during this stage was 56.09 °C for the control sample, while the addition of vegetable purées shifted this peak to 65–76 °C. The mass reduction observed in the control sample was the highest, while evaporation of the compounds with relatively low boiling points from the vegetable films was lower. Such observations lead to the conclusion that the addition of vegetable purées reduced overall moisture content, possibly due to a change in pectin’s hydrophilic nature resulting from the introduction of lipids from the vegetables. On the other hand, the slight changes might have resulted from stronger interactions between water and other matrix compounds, leading to more difficult migration of those volatile compounds [83], thereby elevating the peak decomposition temperature.

The second stage resulted in the greatest weight loss in the samples, which is usually attributed to the decomposition of biopolymer chains, such as polysaccharides and proteins [84]. In the second decomposition stage, the maximum degradation rate occurred at 218.23 °C for the control sample. The incorporation of vegetable purées shifted the decomposition peak (T_max_) to lower temperatures by approximately 25 °C, except for the broccoli-containing film, for which the decrease was only about 7 °C. As reported by Šešlija et al. [85], in pure biopolymer films, this temperature range usually reflects polymer degradation, and the lower peak temperature most likely correlates with the introduction of nutrients (carbohydrates and proteins) into the vegetable purée. Films containing jujube purée also exhibited shifts in the decomposition peak temperature (T_max_), indicating that a higher pectin-to-purée ratio was associated with greater thermal stability and consequently higher decomposition temperatures [86]. The shift in peak temperature may be due to the acidity of the purées, which could catalyze pectin chain scission at lower temperatures. The majority (55%) of the control sample matter, which consisted mainly of citrus pectin and glycerol, degraded at this stage, while the mass of films with the vegetable purées was reduced by over 10%, suggesting a lower pectin and other carbohydrate content. The lowest amount of decomposed matter was observed for the film with the broccoli purée (36.67%).

The third stage was identified between 270 and 600 °C, with the maximum decomposition rate (T_max_) occurring at approximately 318–320 °C for all samples. Although no significant shift in temperature was observed at this stage, the control sample containing only pectin and glycerol showed a very shallow disturbance in the curve, whereas the dTGA curves for the vegetable films exhibited prominent peaks. This was related to the content of the decomposed material, which was considerably smaller in the control sample. According to the report of Burhenne et al. [87], large-molecular compounds of a complex molecular structure, such as cellulose, were probably responsible for this main peak, and after that, the carbonization of the residual matter proceeded, leaving char and ash. Accordingly, the lack of a notable peak in the pure pectin sample was due to no additional polysaccharides originating from vegetable tissue that were incorporated into the film matrix. In the end, the least amount of matter remained after the complete burning of the control sample (24%). At the same time, the inorganic residue in biopolymer films with the addition of vegetable purées ranged from 30.62% to 32.36%. That confirms that the mineral content in vegetable purées was significantly higher, leading to imbalances in the residual mass.

## 3. Materials and Methods

### 3.1. Sample Preparation

The research material consisted of both film-forming solutions and the resulting composite polymer structures in the form of films based on vegetable purées prepared from frozen vegetable outgrades, their blends, and high-methoxyl citrus pectin (E440i), extracted from citrus peels (AGLUPECTIN HS-MR, manufacturer: JRS Silvateam Ingredients S.r.l., Bergamo, Italy). Frozen cauliflower, broccoli, carrot, and pumpkin were used to obtain the vegetable purées. The raw material used to obtain the film-forming solutions and then the composite polymer structures in the form of films was industrially frozen plant by-products, post-calibration waste, supplied by Unifreeze sp. z o.o. (Czarnowo, Poland). All vegetables were stored at −18 °C in tightly sealed plastic zip-lock bags. Glycerol (Avantor Performance Materials Poland S.A., Gliwice, Poland) was used as a plasticizer for the composite polymer film structures.

### 3.2. Technological Methods

Step 1—Preparation of Vegetable Purées

Frozen vegetables were thawed in boiling water, and processing time varied by material: 7 min for broccoli, 10 min for cauliflower, 10 min for pumpkin, and 12 min for carrots. The vegetables were then ground using a knife mill (Grindomix GM 200, Retsch, Katowice, Poland) until smooth. The mill’s operating parameters were adjusted to each vegetable, with the determining factor being the visual assessment of the desired “smoothness” of the resulting purée (Figure 2A). The grinding time and knife speed were adjusted as follows: cauliflower and broccoli—20 s, in the range of 2500–3000 rpm; carrots and pumpkin were ground for 20 s, but the speed was maintained at 3000 rpm.

Step 2—Preparation of Film-forming Solutions Based on Vegetable Purées

Film-forming solutions were prepared from vegetable purées. They consisted of a mixture of distilled water and 5% citrus pectin, which was blended in MSM 7250 (Bosch, Stuttgart, Germany). The semi-finished product was then heated for 30 min and stirred with a magnetic stirrer (RCT basic IKAMAG, IKA Polska, Warsaw) at 400 rpm. After cooling, glycerol was added to the citrus pectin at a 1:2 ratio. The plasticization process was carried out at 50 °C, while stirring the solution continuously. The vegetable purée was mixed with the film-forming solution in a 50:50 weight/weight ratio (Figure 2B). The formulation contained 50% vegetable purée, 2.5% glycerol, 42.5% distilled water, and 5% citrus pectin (Table 9).

**Figure 5:**
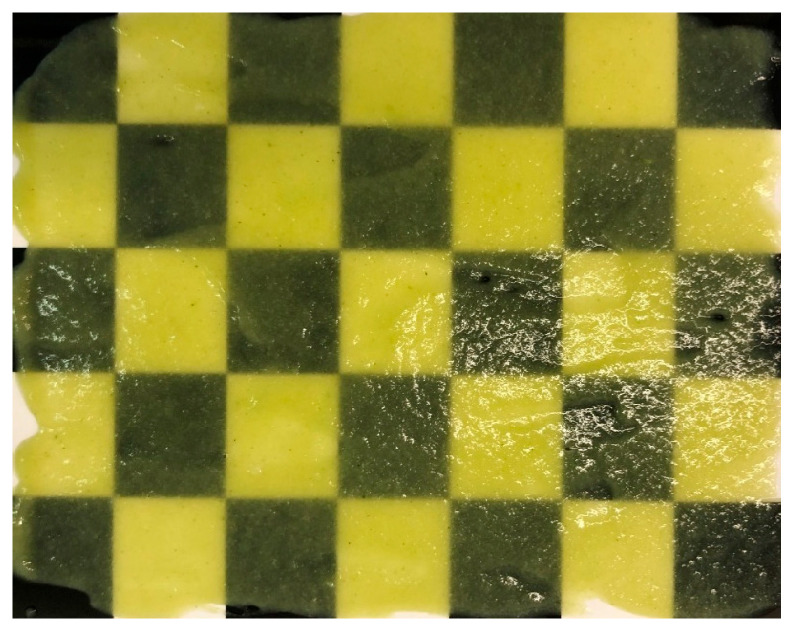
Film-forming solution with the addition of broccoli purée and citrus pectin applied to the byko-chart card.

The prepared film-forming solutions were placed on byko-charts to ensure consistent color and gloss (model A4 PA-2824, Eurotom Sp. z o.o., Poland, Warsaw). The film-forming solution was then spread using an automatic film applicator (ZAA 2300, Zehntner Testing Instruments, Sissach, Switzerland) at a feed speed of 90 mm/s to achieve a thickness of 2500 μm (Figure 5). Film preparation of the specified thickness using the applicator was performed using A4-sized Byko sheets. For the tested film-forming solutions, two sheets of film with a thickness in the range of 2000–2500 μm and dimensions of approximately 20 × 25 cm could be obtained from 100 mL, from which samples were then prepared for individual determinations.

Step 3—Preparation of Films Based on Vegetable Purées

The resulting composite polymer structures in the form of films were dried in a laboratory dryer (SUP-65W, Wamed, Poland, Warsaw) at 60 °C for 6 h and then conditioned in a climate chamber (KBF 720, Binder GmbH, Tuttlingen, Germany) at 50% relative humidity and 25 °C for 48 h. After removing the finished films from the cards, three test samples of each film type were prepared (Figure 1).

### 3.3. Analytical Methods

#### 3.3.1. Properties of Film-Forming Solutions with Vegetable Purées and Citrus Pectin

Rheological properties of the solution

The research was carried out using the MARS 40 oscillatory rheometer by MARS 40 rheometer (ThermoFisher Scientific Inc., Erlangen, Germany). The solutions were tested at 50 °C in a coaxial-cylinder system with a linearly increasing shear rate up to 100 s^−1^. The apparent viscosity of the solution was determined at a shear rate of 50 s^−1^. The Rheowin Job Manager (HAAKE) program was used to register and process the results. Measurements of rheological parameters for the tested film-forming solutions were performed in triplicate.

#### 3.3.2. Properties of Composite Polymer Films with Vegetable Purées and Citrus Pectin

Thickness

Film thickness was measured using a ProGage thickness gauge (Thwing-Albert, Uffenheim, Germany). The measurement was performed with an accuracy of 0.1 μm. Thickness tests for each variant of edible film were conducted in ten repetitions. The applicator ensured consistent wet-film deposition, while the differences in dry-film thickness resulted from the intrinsic solid content and rheological properties of each purée–pectin system.

Water content

On an AE240 analytical balance (Mettler-Toledo International, Inc., Greifensee, Switzerland), 1 g of fragmented films was weighed in weighing bottles with an accuracy of 0.0001 g. The samples prepared in this way were dried in a universal dryer SUP-65 W/G (WAMED Medical Equipment Factory SSP, Warsaw, Poland) at 105 °C until a constant weight. The vials were cooled in a desiccator with CaCl_2_ and weighed again. The assay was performed in triplicate.

Gas permeability

The water vapor permeability was measured gravimetrically, with 50–100% relative humidity differentials using distilled water and a climatic chamber KBF 240 (Binder, GmbH, Tuttlingen, Germany). Circles with a diameter of 3.5 cm were cut out from the obtained film sheets and then placed between two seals located on the neck of a vessel filled halfway with distilled water. Linear regression was used to estimate changes in sample mass over time, omitting the first measurements to stabilize the process conditions. The determination was performed in triplicate. The water vapor permeability was calculated from the formula:

$$P=\frac{∆m\cdot e}{A\cdot ∆t\cdot ∆p}$$

where P—water vapor permeability, (g·(m·s·Pa)^−1^); ∆m·(∆t)^−1^—loss of sample mass over time (g·s^−1^); e—film thickness (m); A—permeation area (8.04^−4^ m^2^); ∆p—pressure difference under and above the film (Pa).

The permeability of oxygen and carbon dioxide was determined by the manometric method according to the ASTM D1434-82 standard [88] using a gas permeability tester model C130 (Labthink Instruments Co., Ltd., Jinan, China). Samples of 10 cm in diameter were cut from each film, and then their thickness was measured and placed in the measuring cell. The measurements were carried out in at least 3 repetitions. The values presented correspond to gas transmission determined under a pressure difference of 0.1 MPa over a 24 h period, which is the standard reporting format provided by the instrument.

Optical properties

Color measurements of the films were performed using a Chroma Meter CR-400 (Konica Minolta Co., Ltd., Japan) in the CIE L*a*b* color system. The films were placed on the measuring plate, and their color was measured 10 times for each film type. After measurement, the films were characterized using the following parameters: total color difference (Δ*E*).

The opacity test was carried out using an Evolution 220 UV–visible spectrometer (Thermo Fisher Scientific, Waltham, MA, USA). The film samples were placed on a magnetic holder and inserted into the device, which measured the absorbance at a wavelength of 600 nm. Ten repetitions were performed for each type of film. For calculations, the equation was used:

$$O=\frac{A_{600}}{l}$$

where O—opacity (a.u./mm); $A_{600}—$absorbance at 600 nm; l—film thickness (mm).

Mechanical properties

Mechanical properties testing was performed using a TA XT2i texturemeter (Stable Micro Systems Ltd., Surrey, UK). The tensile strength was measured according to ASTM D882-12 [89]. A minimum of 10 repetitions was performed for each type of film. Samples measuring 2.5 × 10 cm were cut from the film sheets and then placed between the texture meter jaws, set 25 mm apart. The jaws were moved apart at a speed of 1 m/s. The tensile strength TS and relative elongation E were calculated according to the formulas:

$$TS=\frac{F_{max}}{A}$$

$$E=\frac{∆l}{l_{0}}\cdot 100$$

where $F_{max}$—force causing film rupture (N), A—cross-sectional area of the film before the tensile test, ∆l—elongation of the sample at which the film was broken (mm), $l_{0}$—the initial length of the sample (mm).

Water contact angle

The water contact angle analysis was carried out by applying a drop of distilled water to the surface of the tested edible film. Both air and support surfaces were tested separately. The measurement was performed using an OCA 25 goniometer (DataPhysics Instruments, Filderstadt, Germany). The measurement was performed in 5 repetitions for each type of surface and each type of film. The results were collected and processed using SCA20_U software (Version 5.0.37).

*Microstructure*

The surface and cross-sectional morphologies of the composite films were observed using a HITACHI TM-3000 scanning electron microscope (Hitachi High-Technologies Corporation, Chiyoda, Tokyo, Japan). The samples were coated with gold under vacuum using a Cressington 108 Auto device (Cressington, Watford, UK). Film specimens measuring 5 × 5 mm were attached to the sample mount using carbon tape. An accelerating voltage of 15 kV was applied, and magnifications were selected from 400× to 800×, depending on the objects observed in the images. The presented images show both magnification and distance measurements in micrometers.

Sorption properties

Sorption isotherms were determined using a static method. Weighed samples were placed in a desiccator with a water activity ranging from 0.000 to 0.930. The study was conducted in three replicates for each film. The samples were stored for 3 months at 25 °C. During this time, the samples were weighed every 2 weeks, and after 3 months, their final water activity was determined using a Rotronic HygroLab device according to the manufacturer’s instructions.

The water vapor sorption isotherms of vegetable–pectin composite films were described using the Peleg and GAB mathematical models presented below [82]:
ModelMathematical equationPELEG$u=A\cdot a_{w}^{B}+C\cdot a_{w}^{D}$GAB$u=\frac{u_{m}\cdot k\cdot c\cdot a_{w}}{\left(1-k\cdot a_{w}\right)\cdot \left[1+\left(c-1\right)\cdot k\cdot a_{w}\right]}$

where u is the water content (g H_2_O·g_d.m._^−1^); um is the water content in a monolayer (gH_2_O·g_d.m._^−1^); $a_{w}$ is water activity; *A*, *B*, *C*, *D*, *c*, k are constants.

The fitting of the models was determined by matching experimental and computational data:

RMS—relative mean square error in percentage:

$$RMS=\sqrt{\frac{\sum {\left(\frac{u_{e}-u_{P}}{u_{e}}\right)}^{2}}{n}}\cdot 100\%$$

MRE—mean square error:

$$MRE=\frac{100}{n}\cdot \sum \left|\frac{u_{e}-u_{p}}{u_{e}}\right|$$

SEE—error in estimating the water content:

$$SEE=\sqrt{\sum {\frac{\left(u_{e}-u_{p}\right)}{d_{f}}}^{2}}$$

RSS—sum of residual squares:

$$RSS=\sum {\left(u_{e}-u_{p}\right)}^{2}$$

where $u_{P}$—water content (gH_2_O·g_d.m._^−1^), the indexes mean the data: e—experimental, p—calculated, n—number of data, $d_{f}$—degree of freedom (number of experimental data points *minus number of constants in model)*

*Thermogravimetric analysis*

The thermogravimetric analysis was conducted using a TGA/DSC 3+ instrument (Mettler Toledo, Greifensee, Switzerland). Approximately 6 ± 1 mg of the film was put into an aluminum oxide crucible and heated from 30 to 600 °C in a nitrogen atmosphere with the gas flow rate of 50 mL/minute. The heating rate was 5 °C/minute. Weight loss curves were recorded during the analysis and evaluated using STARe software (version 16.20, Mettler Toledo, Switzerland). Results were expressed as the weight loss and weight loss rate over time of heating curves, as well as peak temperature and weight loss of the main decomposition stages.

#### 3.3.3. Statistical Analysis

All experiments were performed in triplicate, and the results are presented as mean values ± standard deviation. Statistical analyses were carried out using Statistica software (version 13.3, Statsoft Polska Sp. z o. o., Kraków, Poland). The significance of differences among samples was evaluated by one-way analysis of variance (ANOVA) followed by Tukey’s Honestly Significant Difference (HSD; Tukey RIR) post hoc test at a significance level of *p* < 0.05. To comprehensively assess the effect of vegetable puree incorporation on the properties of pectin-based films, the statistical evaluation was conducted in two stages. In the first stage, all samples, including the control pectin film and vegetable-puree-containing films, were compared to determine the overall effect of vegetable puree addition. In the second stage, a detailed analysis was performed considering only the vegetable-puree-based formulations to identify differences attributable to the composition of individual puree systems and to evaluate their contribution to the structuring of the composite pectin films.

Samples sharing the same superscript letter within a row were not significantly different (*p* > 0.05), whereas samples marked with different letters differed significantly (*p* < 0.05). This approach enabled the identification of both the overall effect of vegetable puree incorporation and the specific effects associated with variations in the composition of the vegetable puree formulations.

## 4. Conclusions

The present study demonstrated that vegetable processing by-products derived from broccoli, cauliflower, pumpkin, carrot, and their blends can be successfully utilized as functional components of citrus pectin-based composite edible films. The incorporation of vegetable purées significantly modified the rheological behavior of film-forming solutions and influenced the structural, mechanical, barrier, optical, and thermal properties of the resulting materials.

All vegetable-enriched formulations exhibited stronger non-Newtonian shear-thinning behavior and higher viscosity than the pectin control, indicating the formation of more structured systems due to interactions between citrus pectin and naturally occurring vegetable biopolymers, including pectins, cellulose, hemicelluloses, starch, and proteins. These interactions contributed to the development of thicker films with reduced water content and improved barrier properties against water vapor and carbon dioxide diffusion.

The addition of vegetable components also enhanced the mechanical performance of the films. The carrot-containing film exhibited the highest tensile strength, whereas the pumpkin–carrot formulation showed the most advantageous overall combination of mechanical and barrier properties, including the lowest oxygen permeability, low water vapor permeability, and high tensile strength. In contrast, broccoli- and broccoli–cauliflower-based formulations exerted the strongest effect on rheological properties, producing highly structured film-forming systems with the greatest resistance to flow.

Among the investigated formulations, the pumpkin–carrot composite film appears to be the most promising candidate for future biodegradable packaging applications due to its balanced barrier and mechanical performance. Nevertheless, further studies involving storage tests, food-packaging applications, and long-term stability assessment are required before industrial implementation can be considered.

The obtained results confirm the research hypothesis that vegetable-processing by-products constitute valuable raw materials for the production of composite edible films and that interactions between vegetable-derived biopolymers and citrus pectin improve the mechanical, barrier, and functional properties of the resulting materials. Furthermore, the study demonstrates that the valorization of vegetable by-products can support the development of biodegradable packaging materials consistent with circular economy principles and sustainable food-processing strategies.

## Figures and Tables

**Table 1:** Rheological parameters of film-forming solutions according to Ostwald de Waele regression. The same letters (^a–f^) indicate no statistically significant differences (*p* < 0.05).

Sample	*k* Consistency Coefficient (Pa·s*^n^*)	*n* The Flow Index	*η* Viscosity for Shear Rate $\dot{ɣ}$ (1·s^−1^) ≈ 50	*r^2^* Determination Coefficient
Control	0.289 ± 0.012 ^a^	0.889 ± 0.002 ^f^	0.190 ± 0.009 ^a^	0.999
B	51.38 ± 1.84 ^f^	0.089 ± 0.008 ^a^	1.408 ± 0.036 ^f^	0.648
CF	19.49 ± 0.64 ^d^	0.225 ± 0.014 ^b^	0.928 ± 0.063 ^d^	0.984
P	1.285 ± 0.005 ^ab^	0.595 ± 0.006 ^e^	0.267 ± 0.008 ^a^	0.998
C	14.27 ± 0.64 ^c^	0.260 ± 0.008 ^c^	0.784 ± 0.039 ^c^	0.968
B-CF	41.18 ± 3.42 ^e^	0.092 ± 0.002 ^a^	1.191 ± 0.069 ^e^	0.667
P-C	5.552 ± 0.123 ^b^	0.387 ± 0.004 ^d^	0.530 ± 0.052 ^b^	0.970

Notations: Control—corresponds to the control sample containing only pectin. B—sample with added broccoli purée; CF—sample with added cauliflower purée; P—sample with added pumpkin purée; C—sample with added carrot purée; B-CF—sample with added broccoli and cauliflower purée. P-C—sample with added pumpkin and carrot purée.

**Table 2:** Composition of tested films with vegetable purée and citrus pectin combined with Tukey’s RIR test; variable: dry matter content. Marked (*italic*) differences are significant with *p* < 0.05. The same letters (^a–d^) indicate no statistically significant differences (*p* < 0.05).

Values Calculated Based on Tabulated Data and Determined Dry Matter Content										Thickness
Sample	Water Content (g H_2_O·g_d.m._^−1^)		CARBOHYDRATES (g·(g_d.m._^−1^))			PROTEIN (g·(g_d.m._^−1^))		FAT (g·(g_d.m._^−1^))		(μm)
Control	0.179 ± 0.005 ^e^		0.238 ± 0.018 ^a^			0.001 ± 0.000 ^a^		0.000 ± 0.000 ^a^		125 ± 23 ^a^
B	0.090 ± 0.002 ^a^		0.381 ± 0.006 ^b^			0.154 ± 0.003 ^c^		0.020 ± 0.000 ^b^		282 ± 67 ^d^
CF	0.096 ± 0.001 ^ab^		0.382 ± 0.004 ^b^			0.127 ± 0.003 ^b^		0.010 ± 0.000 ^b^		170 ± 25 ^b^
P	0.099 ± 0.000 ^bc^		0.487 ± 0.000 ^c^			0.054 ± 0.000 ^b c^		0.015 ± 0.000 ^b^		210 ± 32 ^bc^
C	0.105 ± 0.004 ^c^		0.499 ± 0.027 ^c^			0.045 ± 0.003 ^b^		0.010 ± 0.001 ^b^		176 ± 20 ^b^
B-CF	0.095 ± 0.001 ^ab^		0.379 ± 0.004 ^b^			0.140 ± 0.002 ^c^		0.015 ± 0.000 ^b^		252 ± 40 ^cd^
P-C	0.114 ± 0.002 ^d^		0.477 ± 0.012 ^c^			0.048 ± 0.002 ^b^		0.012 ± 0.000 ^b^		180 ± 28 ^b^
**Tukey’s RIR test**										
**The effect of carbohydrate content in the composition of the tested films on the dry matter content**										
		Control M = 0.82098			LC (B, CF, B-CF) M = 0.90669				HC (P, C, P-C) M = 0.89390	
CONTROL					*0.000149*				*0.000149*	
LC (B. CF. B-CF)		*0.000149*							*0.000379*	
HC (P. C. P-C)		*0.000149*			*0.000379*					
**The effect of protein content in the composition of the tested films on the dry matter content**										
		Control M = 0.82098		HP (B, B-CF) M = 0.90790			LP (CF, C, P-C) M = 0.89495		MP (P) M = 0.90109	
Control				*0.000178*			*0.000178*		*0.000178*	
HP (B. B-CF)		*0.000178*					*0.005322*		0.432386	
LP (CF. C. P-C)		*0.000178*		*0.005322*					0.471962	
MP (P)		*0.000178*		0.432386			0.471962			
				HP (B, B-CF) 1.9231			LP (CF, C, P-C) 1.1481		MP (P) 1.1830	
HP (B. B-CF)							*0.000443*		*0.005833*	
LP (CF. C. P-C)				*0.000443*					0.981438	
MP (P)				*0.005833*			0.981438			
**The effect of fat content in the composition of the tested films on the dry matter content**										
		Control M = 0.82098		HF (B, P) M = 0.90571			LF (CF) M = 0.90426		MF (C, B-CF, P-C) M = 0.89536	
Control				*0.000178*			*0.000178*		*0.000178*	
HF (B. P)		*0.000178*					0.990682		0.051581	
LF (CF)		*0.000178*		0.990682					0.256486	
MF (C. B-CF. P-C)		*0.000178*		0.051581			0.256486			
				HF (B, P) 0.27416			LF (CF) 0.09126		MF (C, B-CF, P-C) 0.19602	
HF (B. P)							*0.000178*		*0.000178*	
LF (CF)				*0.000178*					*0.000178*	
MF (C. B-CF. P-C)				*0.000178*			*0.000178*			

Notations: B—broccoli. CF—cauliflower. P—pumpkin. C—carrot. B-CF—broccoli and cauliflower. P-C—pumpkin and carrot. Measurements resulting from the content of individual nutrients: LC—low carbohydrate content. HC—high carbohydrate content; HP—high protein content. LP—low protein content. MP—medium protein content; HF—high fat content. LF—low fat content. MF—medium fat content. Mean values ± standard deviations. Identical letters next to values in columns indicate homogeneous groups and no statistically significant differences (*p* ≤ 0.05).

**Table 3:** Microstructure of the surface and cross-section of composite films obtained based on vegetable purée with citrus pectin.

CONTROL	
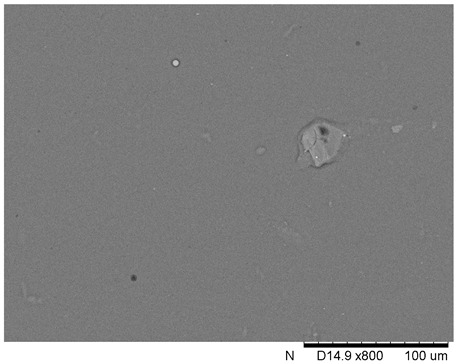	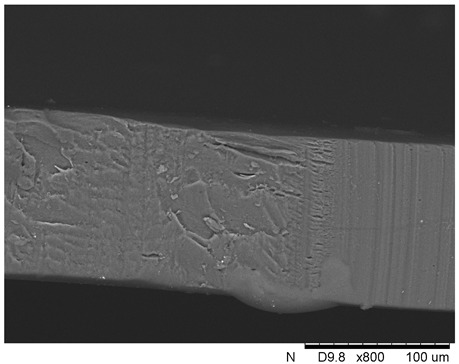
B	
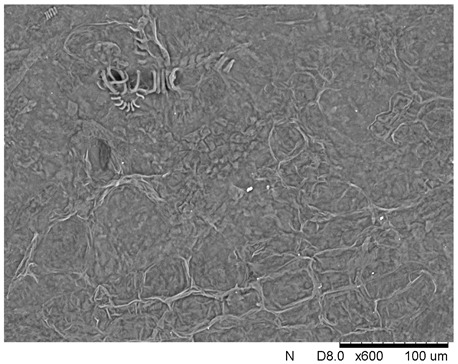	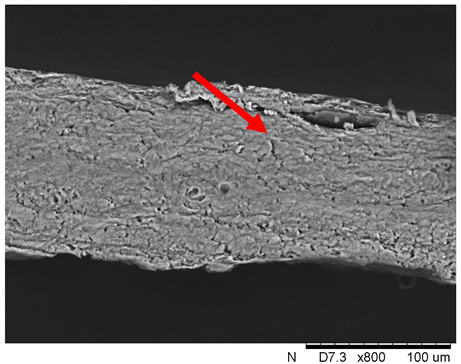
CF	
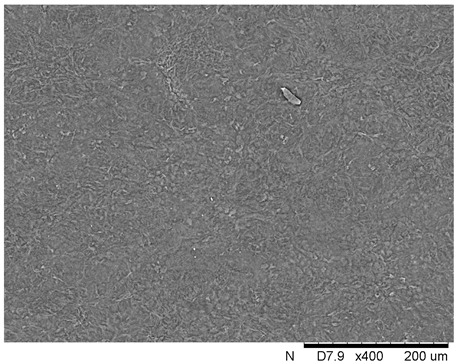	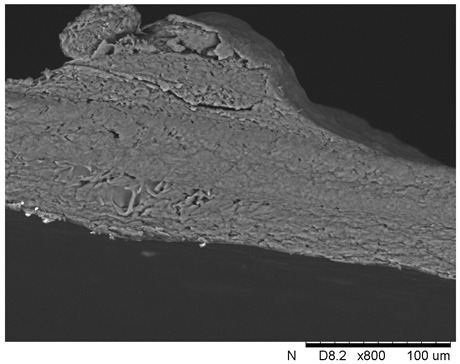
P	
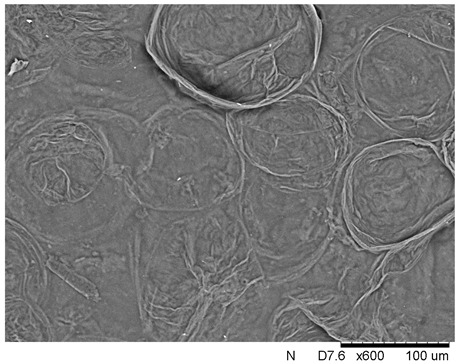	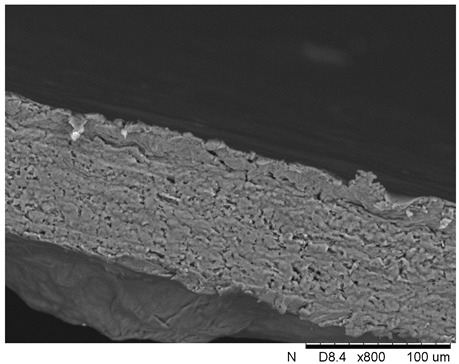
C	
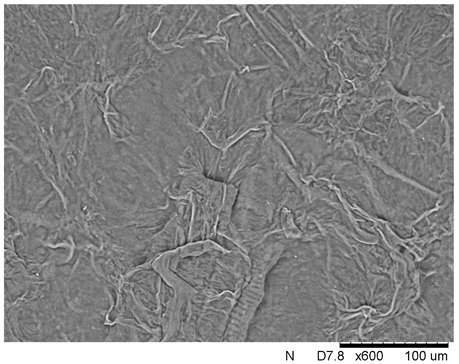	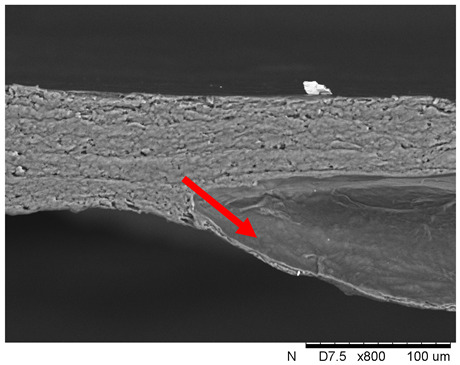
B-CF	
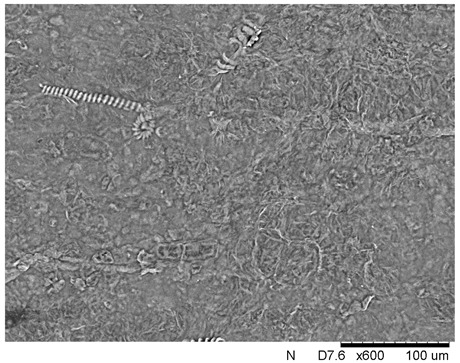	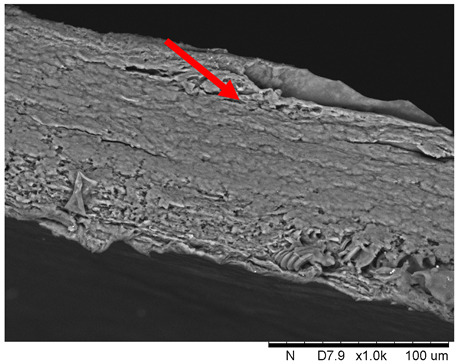
P-C	
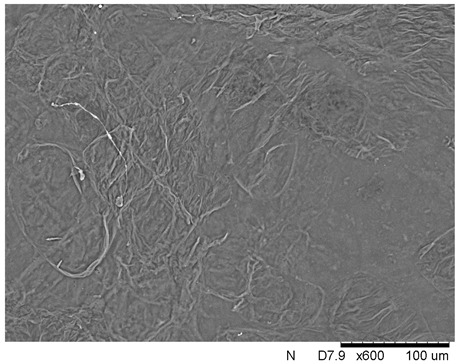	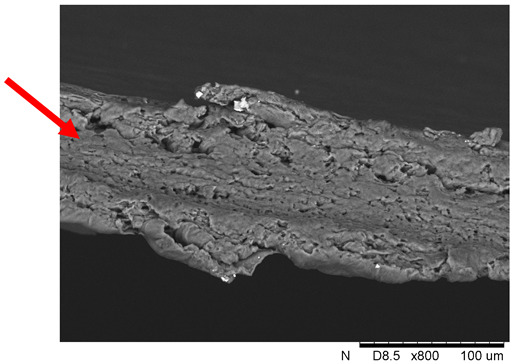

Notations: B—broccoli, CF—cauliflower, P—pumpkin, C—carrot, B-CF—broccoli and cauliflower, P-C—pumpkin and carrot. The arrows indicate the most significant structural changes in the cross-sections of the film matrix.

**Table 4:** Water vapor, oxygen, and carbon dioxide permeability of composite films obtained based on vegetable purée with citrus pectin. Tukey’s RIR test; variable: Water vapor (WVP) and other gases (O_2_ and CO_2_) permeability. The same letters (^a–d^) indicate no statistically significant differences (*p* < 0.05). Marked (*italic)* differences are significant with *p* < 0.05.

Sample	Water Vapor Permeability (10^−10^ g·(m·s·Pa)^−1^)	O_2_ (GRT) (cm^3^·(m^2^·24 h·0.1 MPa)^−1^)			CO_2_ (GRT) (cm^3^·(m^2^·24 h·0.1 MPa)^−1^)
Control	18.99 ± 2.67 ^b^	10.35 ± 0.21 ^ab^			21.95 ± 4.51^b^
B	13.66 ± 1.09 ^a^	39.37 ± 2.37 ^d^			15.02 ± 0.85 ^ab^
CF	13.45 ± 2.71 ^a^	8.20 ± 0.43 ^ab^			17.24 ± 1.35 ^ab^
P	10.74 ± 0.78 ^a^	17.15 ± 1.73 ^ac^			13.62 ± 4.32 ^ab^
C	12.39 ± 0.27 ^a^	15.59 ± 0.52 ^bc^			17.91 ± 0.92 ^ab^
B-CF	14.69 ± 1.03 ^ab^	19.05 ± 6.52 ^c^			10.47 ± 2.26 ^a^
P-C	10.74 ± 0.29 ^a^	6.95 ± 0.55 ^a^			15.50 ± 2.93 ^ab^
**Tukey’s RIR test**					
**The effect of carbohydrate content in the composition of the vegetable–pectin composite films on permeability**					
**O_2_**					
	Control M = 10.349		LC (B, CF, B-CF) M = 22.206		HC (P, C, P-C) M = 12.708
Control			0.196743		0.931801
LC (B, CF, B-CF)	0.196743				0.130665
HC (P, C, P-C)	0.931801		0.130665		
**CO_2_**					
	Control M = 21.984		LC (B, CF, B-CF) M = 14.626		HC (P, C, P-C) M = 15.293
Control			*0.026614*		*0.014612*
LC (B, CF, B-CF)	*0.026614*				0.913660
HC (P, C, P-C)	*0.014612*		0.913660		
**WVP**					
	Control M = 18.987		LC (B, CF, B-CF) M = 13.931		HC (P, C, P-C) M = 11.287
Control			*0.000442*		*0.000150*
LC (B, CF, B-CF)	*0.000442*				*0.005326*
HC (P, C, P-C)	*0.000150*		*0.005326*		
**The effect of protein content in the composition of the tested vegetable–pectin composite films on permeability**					
**O_2_**					
	Control M = 10.349	HP (B, B-CF) M = 11.894		LP (CF, C, P-C) M = 24.669	MP (P) M = 6.9473
Control		0.993429		0.081474	0.957564
HP (B, B-CF)	0.993429			*0.042986*	0.833431
LP (CF, C, P-C)	0.081474	*0.042986*			*0.024440*
MP (P)	0.957564	0.833431		*0.024440*	
**CO_2_**					
	Control M = 21.948	HP (B, B-CF) M = 17.575		LP (CF, C, P-C) M = 14.466	MP (P) M = 15.498
Control		0.224299		*0.009953*	0.085341
HP (B, B-CF)	0.224299			0.259071	0.777141
LP (CF, C, P-C)	*0.009953*	0.259071			0.957438
MP (P)	0.085341	0.777141		0.957438	
**WVP**					
	Control M = 18.987	HP (B, B-CF) M = 12.093		LP (CF, C, P-C) M = 13.578	MP (P) M = 10.736
Control		*0.000382*		*0.001579*	*0.000316*
HP (B, B-CF)	*0.000382*			0.414931	0.707953
LP (CF, C, P-C)	*0.001579*	0.414931			0.116733
MP (P)	*0.000316*	0.707953		0.116733	
**The effect of fat content in the composition of the tested vegetable–pectin composite films on permeability**					
**O_2_**					
	Control M = 10.349	HF (B, P) M = 11.268		LF (CF) M = 15.589	MF (C, B-CF,P-C) M = 22.206
Control		0.999252		0.917866	0.320008
HF (B, P)	0.999252			0.927969	0.203468
LF (CF)	0.917866	0.927969			0.757718
MF (C, B-CF,P-C)	0.320008	0.203468		0.757718	
**CO_2_**					
	Control M = 21.984	HF (B, P) M = 14.319		LF (CF) M = 17.242	MF (C, B-CF,P-C) M = 14.262
Control		*0.028093*		0.366683	*0.025084*
HF (B, P)	*0.028093*			0.634772	0.998263
LF (CF)	0.366683	0.634772			0.671119
MF (C, B-CF,P-C)	*0.025084*	0.998263		0.671119	
**WVP**					
	Control M = 18.987	HF (B, P) M = 10.737		LF (CF) M = 12.388	MF (C, B-CF,P-C) M = 13.931
Control		*0.000179*		*0.000399*	*0.000619*
HF (B, P)	*0.000179*			0.420901	*0.004085*
LF (CF)	*0.000399*	0.420901			0.428164
MF (C, B-CF,P-C)	*0.000619*	*0.004085*		0.428164	

Notations: B—broccoli, CF—cauliflower, P—pumpkin, C—carrot, B-CF—broccoli and cauliflower, P-C—pumpkin and carrot. Measurements resulting from the content of individual nutrients: LC—low carbohydrate content, HC—high carbohydrate content; HP—high protein content, LP—low protein content, MP—medium protein content; HF—high fat content, LF—low fat content, MF—medium fat content. Mean values ± standard deviations. Identical letters next to values in columns indicate homogeneous groups and no statistically significant differences (*p* < 0.05).

**Table 5:** Optical properties of the composite films obtained from vegetable purée with citrus pectin.

Sample	L*	a*	b*	Δ*E*	*O* (a.u.·mm^−1^)
Control	90.09 ± 0.38	−0.77 ± 0.03	3.90 ± 0.07	3.94 ± 0.21	7.10 ± 3.34
B	68.04 ± 1.02	−4.14 ± 0.28	49.28 ± 0.57	54.45 ± 0.94	3.64 ± 1.05
CF	86.82 ± 0.69	−1.23 ± 0.06	18.29 ± 1.70	18.46 ± 1.82	4.66 ± 0.95
P	77.09 ± 2.48	9.75 ± 3.91	83.03 ± 1.73	84.44 ± 2.43	4.05 ± 1.40
C	70.71 ± 0.54	31.69 ± 0.73	61.88 ± 0.11	72.40 ± 0.43	5.53 ± 0.85
B-CF	76.44 ± 0.86	−3.65 ± 0.21	38.12 ± 0.61	40.79 ± 0.66	3.84 ± 0.72
P-C	74.12 ± 1.28	22.34 ± 2.19	71.50 ± 0.70	76.60 ± 1.54	4.54 ± 1.60

Notations: B—broccoli, CF—cauliflower, P—pumpkin, C—carrot, B-CF—broccoli and cauliflower, P-C—pumpkin and carrot.

**Table 6:** Mechanical properties of the composite films obtained from vegetable purée with citrus pectin combined with Tukey’s RIR test; variable: Strength [MPa] and Elongation [%]. The marked (*italic*) differences are significant with *p* < 0.05. The same letters (^a–d^) indicate no statistically significant differences (*p* < 0.05).

Sample	Tensile Strength [MPa]			Elongation [%]
Control	27.38 ± 9.66 ^a^			20.13 ± 5.06 ^d^
B	40.79 ± 15.46 ^ab^			12.79 ± 3.62 ^ab^
CF	46.44 ± 11.69 ^b^			10.15 ± 2.06 ^a^
P	46.28 ± 12.59 ^b^			16.68 ± 3.50 ^cd^
C	62.17 ± 9.60 ^c^			15.86 ± 1.59 ^bc^
B-CF	40. 77 ± 12.10 ^ab^			11.68 ± 2.31 ^a^
P-C	51.02 ± 13.50 ^b c^			15.55 ± 3.77 ^bc^
**Tukey’s RIR test**				
**Carbohydrates content—Strength [MPa]**				
	Control M = 27.384	LC (B, CF, B-CF) M = 43.100		HC (P, C, P-C) M = 52.854
Control		*0.000561*		*0.000105*
LC (B. CF. B-CF)	*0.000561*			*0.002321*
HC (P. C. P-C)	*0.000105*	*0.002321*		
**Carbohydrate content—Elongation [%]**				
	CONTROL M = 20.129	LC (B, CF, B-CF) M = 11.691		HC (P, C, P-C) M = 15.983
Control		*0.000105*		*0.000551*
LC (B. CF. B-CF)	*0.000105*			*0.000106*
HC (P. C. P-C)	*0.000551*	*0.000106*		
**Protein content—Strength [MPa]**				
	Control M = 27.384	HP (B, B-CF) M = 42.664	LP (CF, C, P-C) M = 56.593	MP (P) M = 46.283
Control		*0.000974*	*0.000140*	*0.000864*
HP (B. B-CF)	*0.000974*		*0.000223*	0.786316
LP (CF. C. P-C)	*0.000140*	*0.000223*		0.064847
MP (P)	*0.000864*	0.786316	0.064847	
**Protein content—Elongation [%]**				
	CONTROL M = 20.129	HP (B, B-CF) M = 11.541	LP (CF, C, P-C) M = 15.706	MP (P) M = 16.683
Control		*0.000140*	*0.000748*	*0.038248*
HP (B. B-CF)	*0.000140*		*0.000148*	*0.000154*
LP (CF. C. P-C)	*0.000748*	*0.000148*		0.809525
MP (P)	*0.038248*	*0.000154*	0.809525	
**Fat content—Strength [MPa]**				
	CONTROL M = 27.384	HF (B, P) M = 43.534	LF (CF) M = 46.439	MF (C, B-CF, P-C) M = 51.318
Control		*0.002377*	*0.001843*	*0.000141*
HF (B. P)	*0.002377*		0.912209	0.090301
LF (CF)	*0.001843*	0.912209		0.644777
MF (C, B-CF, P-C)	*0.000141*	0.090301	0.644777	
**Fat content—** **Elongation [%]**				
	CONTROL M = 27.384	HF (B, P) M = 43.534	LF (CF) M = 46.439	MF (C, B-CF, P-C) M = 51.318
Control		*0.000244*	*0.000140*	*0.000148*
HF (B. P)	*0.000244*		*0.001432*	0.974963
LF (CF)	*0.000140*	*0.001432*		*0.001970*
MF (C, B-CF, P-C)	*0.000148*	0.974963	*0.001970*	

**Table 7:** Water contact angle of the films obtained from vegetable purée with citrus pectin, depending on the vegetable input and the air or support side of the tested biopolymer matrix.

Sample/Side	Contact Angle (°)			
	*τ* = 0	*τ* = 30	*τ* = 60	Photo of the Air Side for *τ* = 0
Control				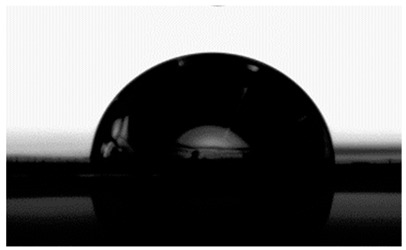
Air	94.11 ± 5.79	90.75 ± 6.77	87.16 ± 5.39	
Support	94.13 ± 5.83	90.80 ± 6.76	87.15 ± 5.40	
B				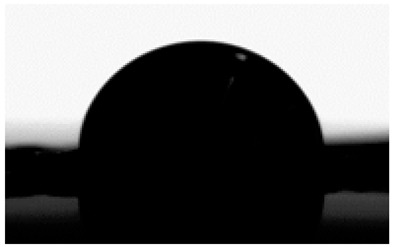
Air	92.13 ± 7.23	80.81 ± 5.29	78.36 ± 4.71	
Support	55.68 ± 6.19	46.43 ± 7.30	38.08 ± 14.02	
CF				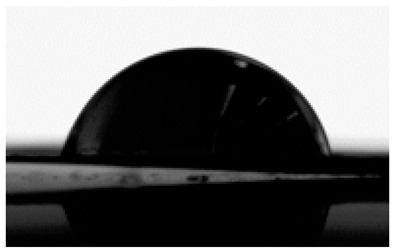
Air	82.95 ± 3.23	77.59 ± 2.91	73.43 ± 4.69	
Support	52.54 ± 5.71	45.99 ± 9.51	36.41 ± 16.93	
P				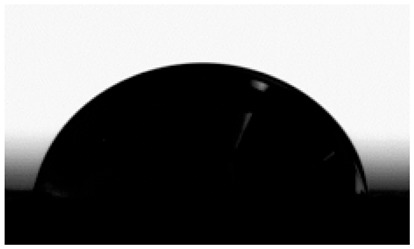
Air	94.83 ± 3.56	76.07 ± 6.55	74.51 ± 6.75	
Support	82.35 ± 6.89	63.30 ± 7.63	62.08 ± 9.82	
C				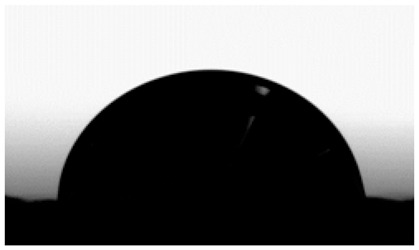
Air	86.00 ± 3.21	73.56 ± 3.98	71.43 ± 3.46	
Support	75.07 ± 9.92	64.70 ± 7.98	63.38 ± 6.50	
B-CF				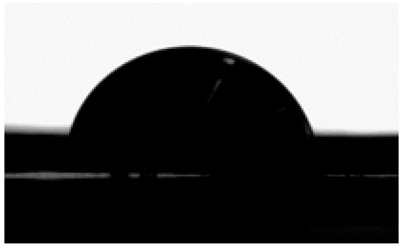
Air	80.70 ± 3.18	76.54 ± 3.14	72.38 ± 5.67	
Support	47.43 ± 3.47	41.12 ± 5.78	34.26 ± 11.44	
P-C				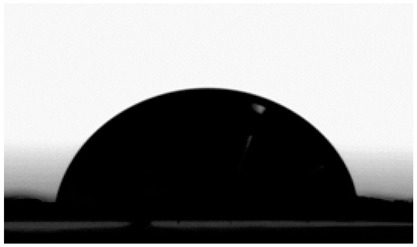
Air	86.43 ± 4.16	66.01 ± 5.12	62.67 ± 8.67	
Support	72.33 ± 2.16	54.60 ± 5.39	55.58 ± 6.87	

**Table 8:** Parameters of thermal decomposition of films with vegetable purée and citrus pectin.

Sample	1st Stage 30–100 °C		2nd Stage 100–270 °C		3rd Stage 270–600 °C	
	Temperature °C	Weight Loss %	Temperature °C	Weight Loss %	Temperature °C	Weight Loss %
Control	56.09	3.70	218.23	55.03	318.70	17.24
B	75.66	2.72	211.23	36.67	318.75	28.73
CF	67.79	3.54	193.96	41.86	320.27	23.91
P	65.81	3.37	193.21	40.38	318.47	25.44
C	70.03	3.01	193.39	42.34	318.90	23.59
B-CF	67.95	2.85	189.93	38.19	319.81	26.6
P-C	68.03	2.87	194.25	41.33	320.19	25.18

Notations: Control—corresponds to the control sample containing only pectin. B—sample with added broccoli purée; CF—sample with added cauliflower purée; P—sample with added pumpkin purée; C—sample with added carrot purée; B-CF—sample with added broccoli and cauliflower purée. P-C—sample with added pumpkin and carrot purée.

**Table 9:** Formulation of film-forming solutions prepared based on vegetable purée and citrus pectin.

Sample	Content of Film-Forming Solution (%)	Contents and Composition of Vegetable Purée (%)
Control	100%	-
B	50%	50% broccoli purée
CF	50%	50% cauliflower purée
P	50%	50% pumpkin purée
C	50%	50% carrot purée
B-CF	50%	25% broccoli purée + 25% cauliflower purée
P-C	50%	25% pumpkin purée + 25% carrot purée

Notations: Control—corresponds to the control sample containing only pectin. B—sample with added broccoli purée; CF—sample with added cauliflower purée; P—sample with added pumpkin purée; C—sample with added carrot purée; B-CF—sample with added broccoli and cauliflower purée. P-C—sample with added pumpkin and carrot purée.

## Data Availability

Data will be available upon reasonable request.
